# Supplementary material for: Enhanced and synergistic catalytic activation by photoexcitation driven S−scheme heterojunction hydrogel interface electric field
Source: Nat Commun. 2023 Oct 23;14:6733. doi: 10.1038/s41467-023-42542-6 (PMC10593843; doi:10.1038/s41467-023-42542-6)
Supplement: Supplementary file 1 — Supplementary Information [file 41467_2023_42542_MOESM1_ESM.pdf]

# *Support Information*

# Enhanced and synergistic catalytic activation by photoexcitation driven S−scheme heterojunction hydrogel interface electric field

Aiwen Wang[1], Meng Du[1], Jiaxin Ni[1], Dongqing Liu[1], Yunhao Pan[1], Xiongying Liang[1], Dongmei Liu[1*], Jun Ma[1], Jing Wang[2,3*] and Wei Wang[1*]

[1]State Key Laboratory of Urban Water Resource and Environment (SKLUWRE), School of Environment, Harbin Institute of Technology, Harbin, 150090, P. R. China

[2]Institute of Environmental Engineering, ETH Zürich, Zürich 8093, Switzerland

[3]Laboratory for Advanced Analytical Technologies, Empa, Swiss Federal Laboratories for Materials Science and Technology, Dübendorf 8600, Switzerland

*Corresponding author: Dongmei Liu, Wei Wang, Jing Wang

*E−mail addresses: ldm819@126.com; wangweirs@hit.edu.cn; jing.wang@ifu.baug.ethz.ch

# Contents

Supplementary Note 1. Chemicals and Reagents ........ 1

Supplementary Note 2 Characterization ........ 1

Supplementary Note 3. Experimental procedures ........ 2

Supplementary Note 4. Computational details ........ 3

Supplementary Figure 1. Schematic illustration of charge–carrier transfer pathways of conventional type–II (a) and S–scheme heterojunction (b). ........ 4

Supplementary Note 5. The advantages of S−scheme heterojunction ........ 4

Supplementary Figure 2. Synthesis schematic illustration (a) and preparation process photo (b) of PBA/$MoS_2$@ chitosan hydrogel and the proportion of the catalyst in each hydrogel sphere. The mass of PBA/$MoS_2$ was divided by the number of spheres to obtain the amount of catalyst per sphere under ideal conditions. ........ 5

Supplementary Figure 3. The SEM images of PBA (a), hierarchical $MoS_2$ (b) and chitosan (the inset of c). The TEM image (c) and photo (d) of PBA/$MoS_2$@CSH. ........ 6

Supplementary Figure 4. X–ray diffraction (XRD) patterns of as–prepared catalysts. ........ 7

Supplementary Figure 5. Catalytic degradation curve of as–prepared catalysts. ........ 8

Supplementary Figure 6. FTIR spectra (a) and EPR spectra (b) of as–prepared catalysts. ........ 9

Supplementary Table 1. The analysis of Fourier Transform Infrared spectroscopy of hybrid composite. ...... 9

Supplementary Figure 7. Catalytic degradation curve of different PMS concentration with (a) and without (b) photoexcitation. ........ 10

Supplementary Table 2. Comparison with other photocatalysts in literature. ........ 11

Supplementary Figure 8. Crystal cell structures of PBA/$MoS_2$ S–scheme heterojunction. ........ 15

Supplementary Figure 9. Kubelka–Munk energy curve plots of as–prepared catalysts. ........ 16

Supplementary Figure 10. Open–circuit potentials (inset) and IEF intensity of PBA@CSH and PBA/$MoS_2$@CSH. (Assuming the intensity of $Bi_2MoO_6$ to be 1). ........ 17

Supplementary Note 6. The internal electric field (IEF) magnitude analysis. ........ 17

Supplementary Figure 11. Photocurrent response curves (a) and EIS Nyquist plots (b) of PBA@CSH and PBA/$MoS_2$@CSH. ........ 18

Supplementary Figure 12. PL characterizations of PBA@CSH and PBA/$MoS_2$@CSH. ........ 19

Supplementary Table 3. Exponential decay–fitted parameters of fluorescence lifetime. ........ 19

Supplementary Table 4. Fitted parameters of the fs-TAS decay curves. ........ 19

Supplementary Figure 13. The high−resolution XPS spectra of Fe 2p after PMS, light or Vis/PMS activation, respectively. ........ 20

Supplementary Figure 14. VB XPS spectra and the corresponding band gap of as–prepared catalysts. ...... 21

Supplementary Figure 15. Catalytic degradation curve of different pH with photoexcitation. ................... 22

Supplementary Figure 16. Crystal cell structures of PMS on PBA/$MoS_2$ and $MoS_2$/PBA. PMS adsorbed at the PBA interface had longer O–O bonds and stronger $E_{ads}$, which indicates that the activation of PMS molecules on Co was more efficient. ........................................................................................ 23

Supplementary Figure 17. FTIR spectra of PBA/$MoS_2$@CSH after cycled.................................................. 24

Supplementary Figure 18. Chronoamperometry (a) and chronopotentiometry (b) curve of PBA/$MoS_2$@CSH with photoexcitation and adding PMS and DC. ........................................................................... 25

Supplementary Figure 19. Catalytic degradation curve of different temperature (a) and DC concentration (b)....................................................................................................................................... 26

Supplementary Figure 20. TOC removal ratio during the degradation of DC by PBA/$MoS_2$@CSH. .............. 27

Supplementary Figure 21. Leakage of Co, Fe and Mo elements into aqueous solution. ............................. 28

Supplementary Table 5. Leakage of ions (Co, Fe and Mo) of catalysts into aqueous solution after photocatalytic degradation of DC. ......................................................................................... 28

Supplementary Table 6. Comparison with other catalysts for the Metal leaching...................................... 29

Supplementary Figure 22. Degradation of three typical antibiotics, sulfonamides (SA, SMX and SDZ), tetracyclines (TC, DC, CTC and OTC) and quinolones (levofloxacin LVF) by PBA/$MoS_2$@CSH, respectively. Reaction Conditions: [concentration] = 20 mg $L^{-1}$, [catalyst] = 5 hydrogel spheres, [PMS] = 0.54 mM. ....... 30

Supplementary Figure 23. Effects of different water matrices (Harbin, China) and anions $Cl^-$, $HCO_3^-$, $NO_3^-$, $SO_4^{2-}$ and HA on the DC degradation efficiencies. ........................................................................ 31

Supplementary Note 7. The degradation efficiency of DC by coexisting inorganic ions .............................. 31

Supplementary Figure 24. Photo of the LED photoexcitation catalytic column equipment. In the simulated continuous flow experiment, 5 mg $L^{-1}$ DC and 0.1g $L^{-1}$ PMS were injected into the top of the device at a flow rate of 2 mL $min^{-1}$ through a peristaltic pump. Samples were collected every 30min when the water flowed out from the bottom of the device. ......................................................................................... 32

Supplementary Figure 25. The recycle of original and cycled XRD patterns (a), XPS spectra (b) and the high−resolution XPS spectra of S 2p (c) and TEM mapping (d) of PBA/$MoS_2$@CSH. The new appearance of the 168.4 eV peak attributed to S (VI) confirmed that $MoS_2$ was involved in the redox reaction and the formation of $SO_4^{2-}$........................................................................................................................ 33

Supplementary Figure 26. Fragment ions and oxidation products in identification of DC in the catalytic system. .................................................................................................................................. 34

Supplementary Figure 27. The HOMO and LUMO of DC. ............................................................................. 35

Supplementary Figure 28. Theoretical calculated developmental toxicity of DC and their degradation intermediates........................................................................................................................ 36

Supplementary Table 7. The structural information of the possible intermediate products. ...................... 37

Supplementary Table 8. Calculated Fukui index of DC. ................................................................................. 38

**Supplementary Note 1.** Chemicals and Reagents

In this work, all reagents used in the experiments were analytical grade without further purification. Cobalt chloride hexahydrate ($CoCl_2·6H_2O$), potassium ferricyanide($K_3[Fe(CN)_6]$), chitosan, doxycycline (DC), Tetracycline (TC), chlortetracycline (CTC), oxytetracycline (OTC) hydrochloride, sulfamethoxazole (SMX), sulfonamide (SA), sulfadiazine (SDZ) and levofloxacin (LVF) were purchased from Aladdin Reagent Company, China. Molybdenum trioxide ($MoO_3$), potassium thiocyanate (KSCN) and sodium citrate ($C_6H_5Na_3O_7$) were purchased from Tianjin Guangfu Fine Chemical Reagent Co. Ltd, China. Absolute ethanol (EtOH), acetic acid ($CH_3COOH$), sodium acetate ($CH_3COONa$) and Sodium hydroxide (NaOH) and was purchased from Tianjin Kermel Chemical Reagent Co. Ltd, China. Deionized water was used throughout all experiments.

**Supplementary Note 2.** Characterization

The crystal structure of as–prepared samples was measured using X–ray diffraction (XRD, Bruker D8 Advance diffractometer, Gobel mirror monochromated Cu Kα radiation, λ = 1.54056 Å). Fourier transform infrared measured (FTIR) by Thermo Nicolet iS50 Spectrometer. Scanning electron microscopy (SEM) was performed with a SIGMA 500 operated at an accelerating voltage of 10 kV. Morphological information was obtained on a Zeiss Neon 40EsV FIBSEM attached with an energy dispersive spectroscopy (EDS). Transmission electron microscopy (TEM) images were obtained with a JEOL 1400 and high–resolution transmission electron microscopy (HRTEM) images were obtained by JEM–2100F instrument with an acceleration voltage of 200 kV. Kelvin probe force microscopy (KPFM) measured by Bruker (Germany) Dimension icon. X–ray photoelectron microscopy (XPS) was acquired on a Thermo Escalab 250 using an Al Kα X–ray source and all the binding energies were calibrated using C 1s peak (284.8 eV). The leakage of elements was tested by the Inductively Coupled Plasma Optical–MassSpectrometry (Agilent 7900). TOC analyzer (5000A, Shimadzu) was used to monitor the total organic carbon (TOC) removal. The zeta potential was measured using a Zeta Sizer Nano–ZS system, Nano Series (Malvern, United Kingdom). The UV/Vis diffuse reflectance spectra were recorded by a spectrophotometer (Shimadzu, UV3600), in which $BaSO_4$ was employed as the background. Photoluminescence (PL) spectroscopy was obtained via the Perkin–Elmer LS–55 spectrofluorometer at room temperature. The time–resolved

fluorescence measurements were recorded on an Edinburgh FLS980 at an excitation wavelength of 365 nm. The electron spin resonance (ESR) analysis was conducted with a Bruke emxplus. Femtosecond transient absorption spectra (TAS) measured by Helios instrument excited at 400 nm laser pulse irradiation. Photoelectrochemical measurements were recorded using an electrochemical workstation (CHI660E, Chenhua) with a standard three–electrode system. The as–prepared photoelectrode, Pt sheet and Ag/AgCl were used as the working electrode, counter electrode and reference electrode, respectively. All photoelectrochemical measurements were conducted in the $Na_2SO_4$ solution (0.5 M). Transformation products (TPs) of DC were analyzed by Waters 2695 with Waters ZQ2000. A volume of 10 μL was injected. A C18 column (4.6 × 250 mm, 3 μm particle size) was applied. The binary mobile phase was comprised of 0.1% formic acid aqueous solution (named as solvent a) and acetonitrile (named as solvent b). The elution process was conducted at a flow rate of 0.6 $mL \cdot min^{-1}$.

**Supplementary Note 3.** Experimental procedures

Photoexcitation synergistic PMS activation of S–scheme heterojunction hydrogels interface was evaluated by degradation of DC in aqueous solution under simulated sunlight (300W xenon lamp, AM1.5G), visible light (420 nm cutoff filter) and LED (5w) light. In a typical degradation test, five hydrogel spheres were dispersed in 30 mL DC aqueous solution, then a certain amount of PMS was added. At regular intervals, 2mL solution was removed and filtered through a 0.22μm polyether sulfone (PES) filter. Then the concentration of residual DC in the reaction solution was immediately determined by UV–visible spectrophotometer at a wavelength of 346 nm. Contrast the synergies of light excitation with and without light

The used hydrogel spheres were collected and washed with deionized water and ethanol to remove residual organics for repeatability and stability testing. Experiments were performed at an initial pH 5.76 to evaluate catalytic performance. The initial pH of the reaction solution was not adjusted unless otherwise stated. The desired pH of solutions was achieved by adding 1 mM NaOH or 1 mM $H_2SO_4$ and measured using a pH meter. Each set of experiments was performed in triplicate and results are reported as the mean of the triplicates. In addition, error bars indicate the reproducibility of data from repeated experiments. The main oxidative species detected by the trapping experiments were $\cdot O_2^-$, $\cdot OH$, $SO\cdot_4^-$, holes and $^1O_2$ by using L–ascorbic acid, TBA, MeOH EDTA–2Na and

furfural, respectively.

**Supplementary Note 4.** Computational details

Density functional theory (DFT) calculation was carried out by Material Studio software packages were used to study the electron density difference and the density of states (DOS), and carried out by CASTEP module within the plane–wave pseudopotential method. The exchange–correlation function was modeled using the generalized gradient approximation (GGA) with Perdew–Burke–Ernzerh (PBE). For the plane–wave basis set, a cutoff of $E_{cut}$ = 517 eV and a 3×3×3 Monkhorst–Pack k–point mesh has been used for the energy calculations. Structural optimization using super–soft pseudopotentials to describe the interaction of valence electrons and nuclei. The convergence criterion for geometric structure optimization is: the convergence thresholds for atomic displacement, interatomic forces, and interatomic internal stress were taken as 0.001 Å, 0.03 eV/Å, and 0.05 GPa, respectively. The geometric optimization, Fukui function and molecular orbital were performed using DMol3 program B3LYP with DNP 3.5 basis set and COSMO's water solvation model. The frontier electron densities of the highest occupied molecular orbital (HOMO) and the lowest unoccupied molecular orbital (LUMO) were used for predicting the molecular reaction sites that the reactive species easily attacked in catalytic system.

The adsorption energy was calculated via the following Eq. (S2).

$$E_{ads}=E_{tot}\,(\text{Substrate+molecule})–E_{tot}\,(\text{Substrate})–E_{tot}\,(\text{molecule}) \qquad \text{(S2)}$$

where $E_{tot}$ (Substrate+molecule) and $E_{tot}$ (Substrate) were the total energies of PBA/$MoS_2$ with and without $HSO_5^-$ molecule. $E_{tot}$ (molecule) was the energy of $HSO_5^-$.

With regards to the definition of adsorption energy, negative values denote the favorable adsorption site, while positive results indicate it requires energy to adsorption a small molecular on the surface. Generally, the larger the bond order of a molecule, the more stable it is, the less likely it is to react. Besides, Fukui function is a very important concept in conceptual density functional theory, which has been widely used for regioselective prediction of electrophilic, nucleophilic, and radical attacking.

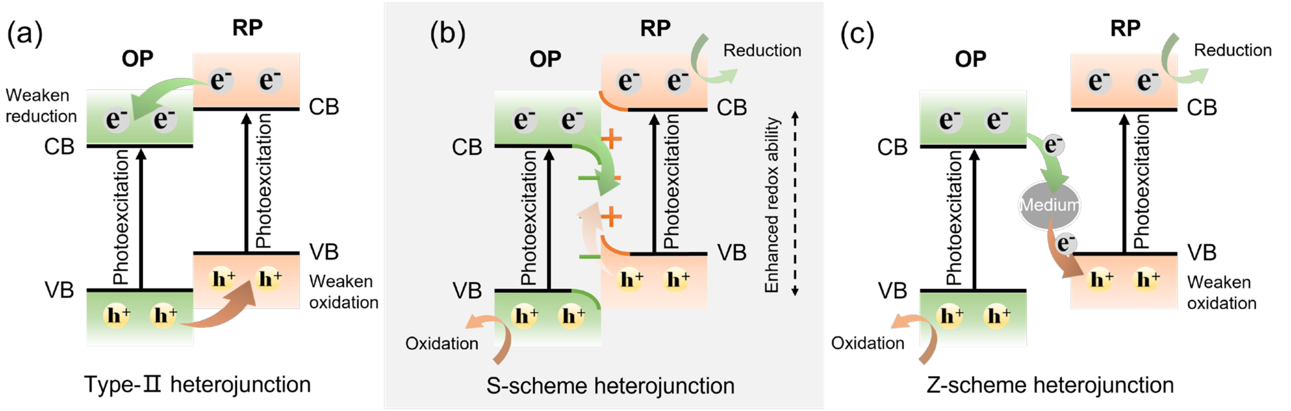


**Supplementary Figure 1. Schematic illustration of charge–carrier transfer pathways. a** Type–II, **b** S–scheme and **c** Z–scheme heterojunction.

**Supplementary Note 5. The advantages of S−scheme heterojunction**

The S−scheme heterojunction is composed of reduced semiconductor photocatalyst (RP) and oxidized semiconductor photocatalyst (OP) with a staggered band structure, which is similar to the conventional type–II heterojunction but has completely different charge transfer routes. For type–II heterojunction, the photo generated electrons of the RP with higher CB are transferred from the interface to the OP with lower CB, while the photo generated holes of the OP with lower VB migrate to the RP with higher VB, resulting in a decrease in the redox ability of the system (Supplementary Figure 1a). On the contrary, the S−scheme heterojunction photogenerated carriers accumulate on the RP at higher CB positions and the OP at lower VB positions, thereby enhancing the charge separation and redox ability of the charge carriers (Supplementary Figure 1b). Therefore, compared with the traditional type I and type II heterostructures, the S−scheme heterojunction improves the redox ability, and compared with the Z–type heterostructure, it does not need the medium of electron transfer at the interface (Supplementary Figure 1c).

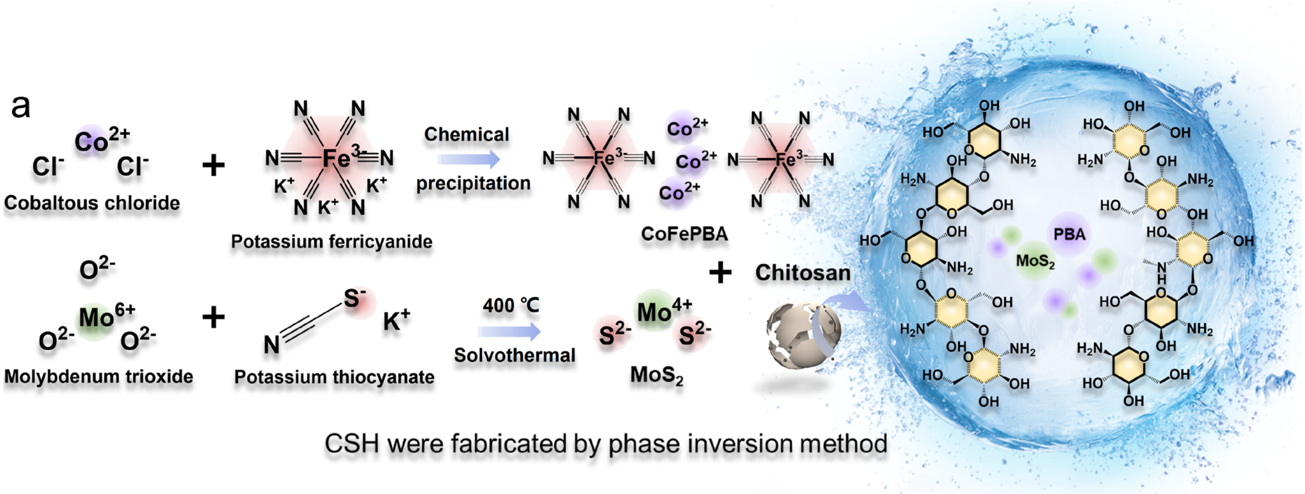


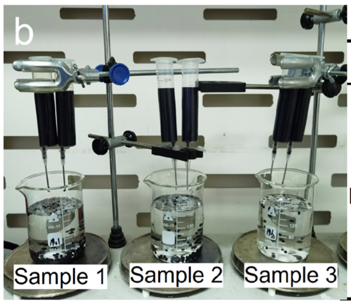


c Catalyst proportion

| | Sample 1 | Sample 2 | Sample 3 | Average |
|---|---|---|---|---|
| Hydrogel spheres | 1778 | 1783 | 1779 | 1777 |
| Chitosan addition | 1.00 g | 1.00 g | 1.00 g | 1.00 g |
| PBA/$MoS_2$ (1:1) addition | 0.40 g | 0.40 g | 0.40 g | 0.40 g |
| PBA/$MoS_2$ per ball | 0.23 mg | 0.22 mg | 0.23 mg | 0.23 mg |
| Chitosan per ball | 0.56 mg | 0.56 mg | 0.57 mg | 0.56 mg |

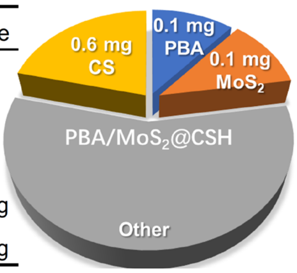


**Supplementary Figure 2. Synthesis methods and details. a, b** Synthesis schematic illustration and preparation process photo of PBA/$MoS_2$@ chitosan hydrogel and the proportion of the catalyst in each hydrogel sphere. **c** The mass of PBA/$MoS_2$ was divided by the number of spheres to obtain the amount of catalyst per sphere under ideal conditions.

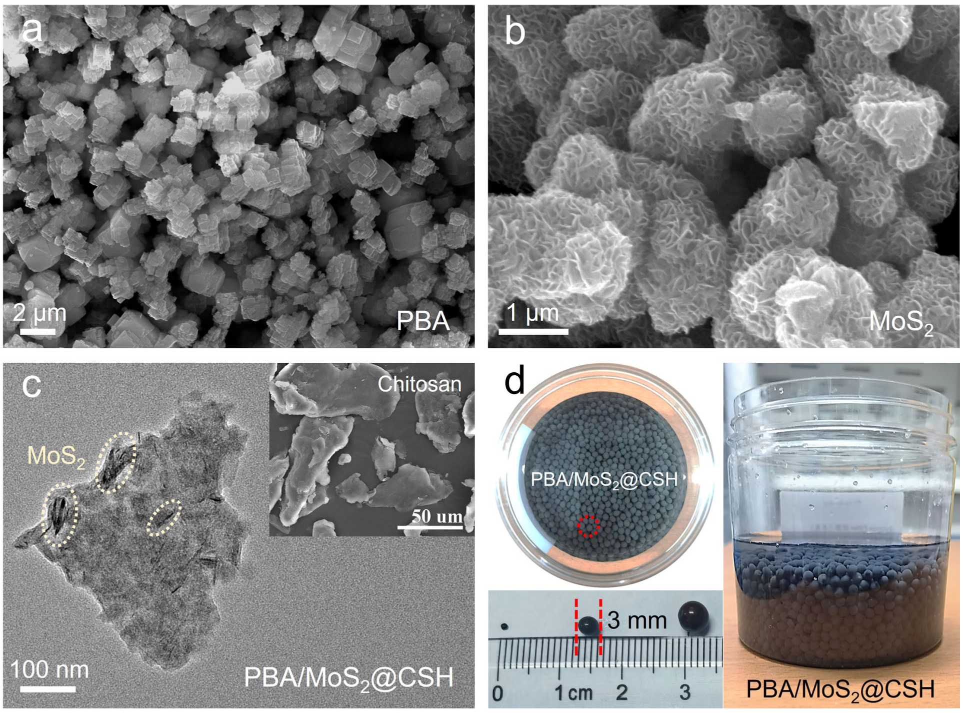


**Supplementary Figure 3. Structural characterization of synthetic materials. a** The SEM images of PBA, **b** hierarchical $MoS_2$ and chitosan (**the inset of c**). **c** The TEM image and **d** photo of PBA/$MoS_2$@CSH.

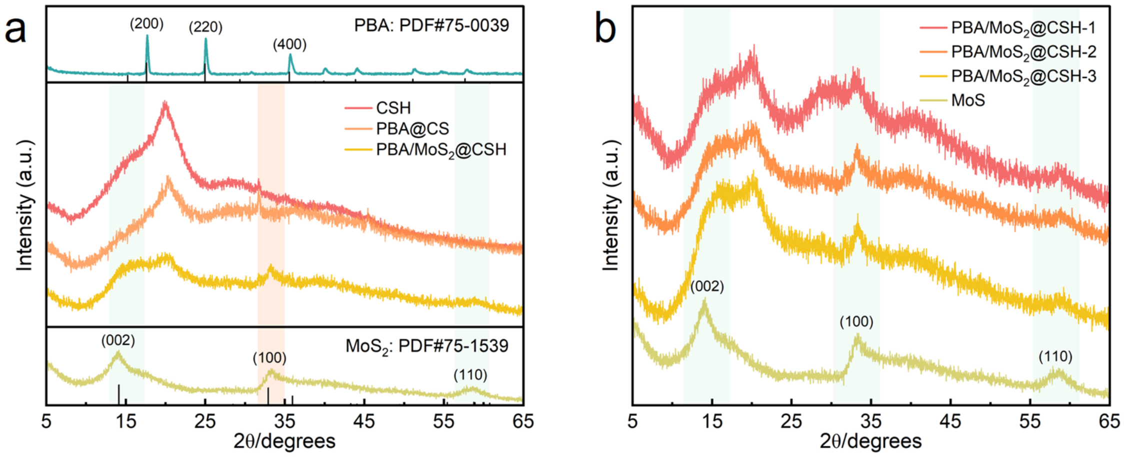


**Supplementary Figure 4. X–ray diffraction (XRD) patterns** of as–prepared catalysts, highlight indicate the positions of crystallization.

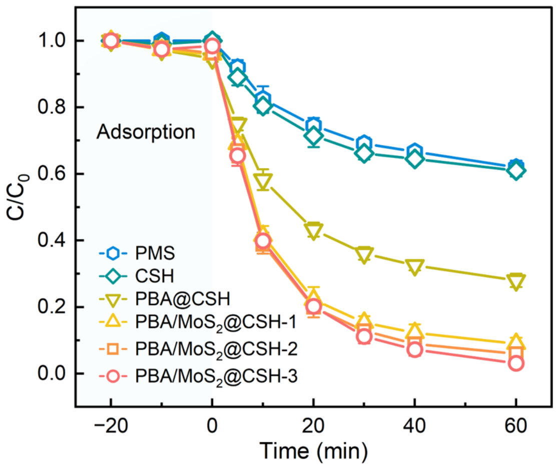


**Supplementary Figure 5. Catalytic degradation curve** of as–prepared catalysts. Error bars represent the standard deviation of the experiment in triplicate.

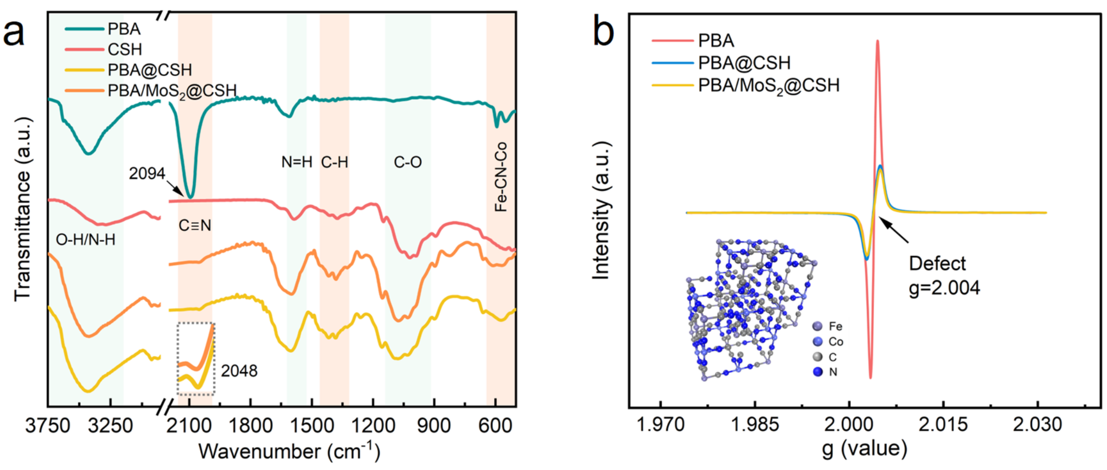


**Supplementary Figure 6. FTIR spectra (a) and EPR spectra (b)** of as–prepared catalysts, highlight indicate the positions of functional group.

**Supplementary Table 1.** The analysis of Fourier Transform Infrared spectroscopy of hybrid composite.

| Wavenumber ($cm^{-1}$) | Characteristic Group |
|---|---|
| 3404−3386 | The stretching vibration of O−H and N−H |
| 2932/2870 | The stretching vibration of C−H |
| 2094/2048 | The stretching vibration of C≡N |
| 1657 | The stretching vibration of amide groups I (C=O), |
| 1586 | The bending vibration of N−H (Including amide groups II) |
| 1460/1417/1383 | The bending vibration of alkyl group |
| 1151 | The stretching vibration of C−O−C |
| 1070 | The stretching vibration of $C_3$−OH (Secondary hydroxyl) |
| 1029 | The stretching vibration of $C_6$−OH (Primary hydroxyl) |
| 595 | The absorption peaks of Fe–CN |
| 553 | The absorption peaks of Fe–CN–Co |

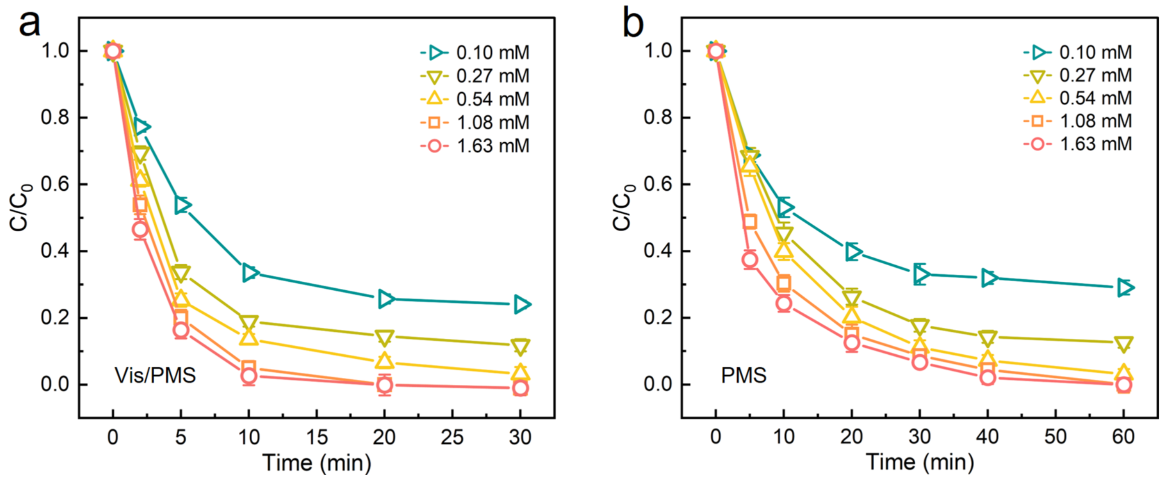


**Supplementary Figure 7. Catalytic degradation curve**. Different PMS concentration with (**a**) and without (**b**) photoexcitation. Error bars represent the standard deviation of the experiment in triplicate.

**Supplementary Table 2.** Comparison with other photocatalysts in literature.

| Photocatalyst | Pollutant | Light source | PMS | Dosage | Removal Time | k ($min^{-1}$) | Reference |
|---|---|---|---|---|---|---|---|
| $PBA/MoS_2@CSH$ | Doxycycline (20 mg $L^{-1}$) | 300W XL (λ ≥ 420 nm) | 0.333 g $L^{-1}$ | 0.038 g $L^{-1}$ | 97.02% 30 min | 0.1147 | This study |
| $LaFeO_3/SBA–15$ | Doxycycline (40 mg $L^{-1}$) | 300 W XL (λ ≥ 420 nm) | 0.615 g $L^{-1}$ | 0.500 g $L^{-1}$ | / | 0.023 | [2] |
| $BiFeO_3/SBA–15$ | Doxycycline (40 mg $L^{-1}$) | 300 W XL (λ ≥ 420 nm) | 1.844 g $L^{-1}$ | 0.500 g $L^{-1}$ | / | 0.0175 | [2] |
| $BiO_{1-x}Cl$ | Doxycycline (50 mg $L^{-1}$) | 5 W LED (λ ≥ 400 nm) | 0.250 g $L^{-1}$ | 0.500 g $L^{-1}$ | 79.4 105 | 0.0062 | [3] |
| $g–C_3N_4/Na–BiVO_4$ | Tetracycline (20 mg $L^{-1}$) | 300 W XL (λ ≥ 420 nm) | 0.307 g $L^{-1}$ | 0.200 g $L^{-1}$ | 98.2% 40 min | 0.109 | [4] |
| $Bi_2MoO_6/CuWO_4$ | Tetracycline (10 mg $L^{-1}$) | 300 W XL (λ ≥ 420 nm) | 0.200 g $L^{-1}$ | 0.200 g $L^{-1}$ | 84.6% 20 min | 0.121 | [5] |
| $10\%Co_3O_4/g–C_3N_4$ | Tetracycline (20 mg $L^{-1}$) | 300 W XL (λ ≥ 420 nm) | 0.061 g $L^{-1}$ | 0.200 g $L^{-1}$ | 98% 60 min | 0.079 | [6] |
| BC/CN–15 | Tetracycline (10 mg $L^{-1}$) | 300 W XL (λ ≥ 420 nm) | 0.200 g $L^{-1}$ | 0.200 g $L^{-1}$ | 90% 60 min | 0.035 | [7] |
| Cu–R | Tetracycline (30 mg $L^{-1}$) | 300 W XL (λ ≥ 420 nm) | 0.300 g $L^{-1}$ | 0.200 g $L^{-1}$ | 96% 60 min | 0.046 | [8] |
| $MnCo_2O_4$ | Tetracycline (30 mg $L^{-1}$) | 300 W XL (λ ≥ 420 nm) | 0.750 g $L^{-1}$ | 0.200 g $L^{-1}$ | 98% 60 min | 0.052 | [9] |
| $3NiO/g–C_3N_4$ | Tetracycline (20 mg $L^{-1}$) | 300 W XL (λ ≥ 420 nm) | 0.100 g $L^{-1}$ | 0.200 g $L^{-1}$ | / 60 min | 0.080 | [10] |
| MCN | Tetracycline (10 mg $L^{-1}$) | 300W XL (λ ≥ 420 nm) | 0.500 g $L^{-1}$ | 0.400 g $L^{-1}$ | 89.7 30 | 0.061 | [11] |
| $MoS_2/Ag/g–C_3N_4$ | Tetracycline (20 mg $L^{-1}$) | 300 W XL (λ ≥ 420 nm) | 0.061 g $L^{-1}$ | 0.200 g $L^{-1}$ | 91.2% 30 min | 0.084 | [12] |
| $MoO_3/Bi_2O_3/g–C_3N_4$ | Tetracycline (40 mg $L^{-1}$) | Solar light | 2.459 g $L^{-1}$ | 0.600 g $L^{-1}$ | 98% 120min | 0.0248 | [13] |
| CuHNPs–7.5 | Tetracycline (40 mg $L^{-1}$) | 100W LED (λ≥ 420 nm) | 0.277 g $L^{-1}$ | 0.200 g $L^{-1}$ | 97.8% 30 min | 0.125 | [14] |
| $FeMo_3O_x/C_3N_4–EP$ | Oxytetracycline (50 mg $L^{-1}$) | 300 W XL (λ ≥ 420 nm) | 3.074 g $L^{-1}$ | 1.000 g $L^{-1}$ | 98.1 | 0.181 | [15] |

| Photocatalyst | Pollutant | Light source | PMS | Dosage | Removal Time | k ($min^{-1}$) | Reference |
|---|---|---|---|---|---|---|---|
| SMM–3 | Levofloxacin (10 mg·$L^{-1}$) | 300 W XL (λ ≥ 420 nm) | 0.500 g $L^{-1}$ | 0.100 g $L^{-1}$ | 95.1% 20 min | 0.196 | [16] |
| Ag/AgCl@ZIF–8/g–$C_3N_4$ | Levofloxacin (10 mg·$L^{-1}$) | 150 W XL (λ ≥ 420 nm) | 1.230 g $L^{-1}$ | 1.000 g $L^{-1}$ | 87.3% 60 min | 0.03054 | [17] |
| $Fe_3O_4$@$CeO_2$@BiOI | Sulfamethoxazole (13 mg·$L^{-1}$ ) | UVA–LED | 0.123 g $L^{-1}$ | 0.100 g $L^{-1}$ | 97% 15 min | 0.221 | [18] |
| γ–$Fe_2O_3$–$MnO_2$ | Ciprofloxacin (17 mg·$L^{-1}$) | 300 W XL (λ ≥ 420 nm) | 0.300 g $L^{-1}$ | 0.150 g $L^{-1}$ | 98.3% 30 min | 0.114 | [19] |
| $CoCr_2O_4$/α–$Fe_2O_3$/β–$La_2S_3$ | Doxycycline (10 mg $L^{-1}$) | 1000W HL (λ ≥ 420 nm) | / | 0.050 g $L^{-1}$ | 92.83% 345 min | 0.0076 | [20] |
| Co/Mn–MOF–74@g–$C_3N_4$ | Doxycycline (40 mg $L^{-1}$) | 300 W XL (λ ≥ 420 nm) | / | 0.500 g $L^{-1}$ | / | 0.00459 | [21] |
| g–$C_3N_4$/α–$Bi_2(MoO_4)_3$ | Doxycycline (10 mg $L^{-1}$) | 500 W XL (λ ≥ 420 nm) | / | 1.000 g $L^{-1}$ | 93.19% 140 min | 0.0183 | [22] |
| $In_2O_3$/$Bi_4O_7$ | Doxycycline (20 mg $L^{-1}$) | 300W XL (λ ≥ 420 nm) | / | 0.500 g $L^{-1}$ | 92.1% 120 min | 0.0197 | [23] |
| AN@CN | Doxycycline (50 mg $L^{-1}$) | 300W XL (λ ≥ 420 nm) | / | 0.500 g $L^{-1}$ | 98.67% 60 min | 0.04052 | [24] |
| BiM/ZnC@PANI | Doxycycline (10 mg $L^{-1}$) | 300W XL (λ ≥ 420 nm) | / | 0.100 g $L^{-1}$ | 90% 150 min | 0.0119 | [25] |
| ILDAc/MIL–68(In)–NH2 | Doxycycline (10 mg $L^{-1}$) | 500 W XL (λ ≥ 420 nm) | / | 0.200 g $L^{-1}$ | 92% 180 min | 0.00918 | [26] |
| ZnO | Doxycycline (10 mg $L^{-1}$) | 30 W UV–C lamp | / | 0.250 g $L^{-1}$ | ~88% 780 min | 0.012 | [27] |
| Bi7O9I3/g–C3N4 | Doxycycline (20 mg $L^{-1}$) | 300W XL (λ ≥ 420 nm) | / | 0.500 g $L^{-1}$ | 80% 120 min | 0.0125 | [28] |
| BiOBr/$FeWO_4$ | Doxycycline (20 mg $L^{-1}$) | 300 W XL (λ ≥ 420 nm) | / | 1.000 g $L^{-1}$ | 90.4% 60 % | 0.0375 | [29] |
| Nd–$BiO_{2-x}$ | Doxycycline (10 mg $L^{-1}$) | 300 W XL (λ ≥ 420 nm) | / | 0.200 g $L^{-1}$ | 86.14% 120 min | 0.01344 | [30] |
| $Co_3O_4TiO_2$/GO | Oxytetracycline (10 mg $L^{-1}$) | 300 W XL (λ ≥ 400 nm) | / | 0.250 g $L^{-1}$ | 91% 90 min | 0.0272 | [31] |
| Ag/p–$Ag_2S$/n–BiVO4 | Oxytetracycline (20 mg $L^{-1}$) | 500 W XL (λ ≥ 420 nm) | / | 0.400 g $L^{-1}$ | 99.8% 150 min | 0.0411 | [32] |
| AgI/BiVO4 | Oxytetracycline | 300 W XL (λ ≥ 420 nm) | / | 0.600 g $L^{-1}$ | 80% | 0.0239 | [33] |

| Photocatalyst | Pollutant | Light source | PMS | Dosage | Removal Time | k ($min^{-1}$) | Reference |
|---|---|---|---|---|---|---|---|
| | (20 mg $L^{-1}$) | | | | 60 min | | |
| SrTiO3/BiOI | Oxytetracycline (20 mg $L^{-1}$) | 300 W XL (λ ≥ 420 nm) | / | 1.000 g $L^{-1}$ | 85.34% 90 min | 0.0252 | 34 |
| Ag/N–GQDs/g–$C_3N_4$ | Tetracycline (10 mg $L^{-1}$) | 300 W XL (λ ≥ 365 nm) | / | 0.200 g $L^{-1}$ | 92.8% 60 min | 0.0428 | 35 |
| CQDs/g–$C_3N_4$ | Tetracycline (10 mg $L^{-1}$) | 250 W XL (λ ≥ 420 nm) | / | 0.500 g $L^{-1}$ | 78.6% 240 min | 0.00642 | 36 |
| h–BN/g–$C_3N_4$ | Tetracycline (10 mg $L^{-1}$) | 300 W XL (λ ≥ 420 nm) | / | 1.000 g $L^{-1}$ | 79.7% 60 min | 0.02775 | 37 |
| h–BN/$Bi_2MoO_6$ | Tetracycline (20 mg $L^{-1}$) | 300W XL (λ ≥ 420 nm) | / | 0.500 g $L^{-1}$ | 99.19% 140 min | 0.0273 | 38 |
| NGQDs–BiOI/$MnNb_2O_6$ | Tetracycline (10 mg $L^{-1}$) | 250 W XL (λ ≥ 420 nm) | / | 0.500 g $L^{-1}$ | 87.2% 60 min | 0.0331 | 39 |
| $TiO_2$@$V_2O_5$–PPy | Tetracycline (50 mg $L^{-1}$) | 300W XL (λ ≥ 420 nm) | / | 0.600 g $L^{-1}$ | 96% 60 min | 0.04498 | 40 |
| Mg–Fe LDH@bioachar | Doxycycline (35 mg $L^{-1}$) | / | 0.750 g $L^{-1}$ | 0.750 g $L^{-1}$ | 88.76% 120 min | 0.23571 | 41 |
| MnO/CoO/$WO_3$ | Doxycycline (20 mg $L^{-1}$) | / | 0.100 g $L^{-1}$ | 0.500 g $L^{-1}$ | 80.04% 120 min | 0.0471 | 42 |
| CuO/$Fe_2O_3$ | Doxycycline (50 mg $L^{-1}$) | | 0.050 g $L^{-1}$ | 0.200 g $L^{-1}$ | 92.6% 120 min | 0.04342 | 43 |
| $FeVO_4$ nanorods | Oxytetracycline (20 mg $L^{-1}$) | | 0.615 g $L^{-1}$ | 0.800 g $L^{-1}$ | 100% 30 min | 0.107 | 44 |
| Co–Fe/$NC^{0.7}$@GCS | Sulfamethoxazole (20 mg·$L^{-1}$) | | 0.0615 g $L^{-1}$ | 0.200 g $L^{-1}$ | 90.2% 60 min | 0.072 | 45 |
| NSC–3 | Sulfamethoxazole (20 mg·$L^{-1}$) | | 0.307 g $L^{-1}$ | 0.200 g $L^{-1}$ | 98.62% 90 min | 0.058 | 46 |
| MF | Sulfamethoxazole (10 mg·$L^{-1}$) | | 0.492 g $L^{-1}$ | 0.060 g $L^{-1}$ | 100% 80min | 0.050 | 47 |
| BC700Fe20 | Sulfamethoxazole (10 mg·$L^{-1}$) | | 1.230 g $L^{-1}$ | 0.500 g $L^{-1}$ | 82.2% 120min | 0.031 | 48 |
| $Co_3O_4$/CPANI | Tetracycline (20 mg $L^{-1}$) | | 0.150 g $L^{-1}$ | 0.150 g $L^{-1}$ | 92.11% 40 min | 0.09033 | 49 |
| $Fe_3O_4$@PANI–p | Tetracycline (20 mg $L^{-1}$) | | 2.459 g $L^{-1}$ | 0.400 g $L^{-1}$ | 89.8% 90 min | 0.0353 | 50 |

| Photocatalyst | Pollutant | Light source | PMS | Dosage | Removal Time | k ($min^{-1}$) | Reference |
|---|---|---|---|---|---|---|---|
| PFSC–900 | Tetracycline (20 mg $L^{-1}$) | | 0.300 g $L^{-1}$ | 0.400 g $L^{-1}$ | 90.91% 120 min | 0.0317 | [51] |
| Goethite/biochar | Tetracycline (30 mg $L^{-1}$) | | 0.615 g $L^{-1}$ | 0.050 g $L^{-1}$ | 72.99% 60 min | 0.02062 | [52] |
| Co–Ni LDO | Tetracycline (30 mg $L^{-1}$) | | 0.984 g $L^{-1}$ | 0.100 g $L^{-1}$ | 100% 60 min | ~0.06 | [53] |
| $CoFe_2O_4$ | Levofloxacin (5 mg·$L^{-1}$) | | 0.154 g $L^{-1}$ | 0.100 g $L^{-1}$ | 94.63% 30 min | 0.0997 | [54] |
| CA–LDH | Ciprofloxacin (20 mg $L^{-1}$) | | 0.500 g $L^{-1}$ | 0.200 g $L^{-1}$ | 98% 60 min | 0.088 | [55] |
| Co@N–BC | Doxycycline (50 mg $L^{-1}$) | | 0.307 g $L^{-1}$ | 0.400 g $L^{-1}$ | 92.72% 30 min | 0.0873 | [56] |
| Cu–$In_2O_3/O_v$ | Tetracycline (20 mg $L^{-1}$) | | 0.300 g $L^{-1}$ | 0.500 g $L^{-1}$ | 100% 20 min | 0.2648 | [57] |
| EGCG@$Fe_3O_4$ | Sulfadiazine (10 mg $L^{-1}$) | | 0.184 g $L^{-1}$ | 0.800 g $L^{-1}$ | 97.9% 60 min | 0.0541 | [58] |
| $SBC_{800}$ | Norfloxacin (10 mg $L^{-1}$) | | 0.307 g $L^{-1}$ | 0.200 g $L^{-1}$ | 100% 40 min | 0.0785 | [59] |

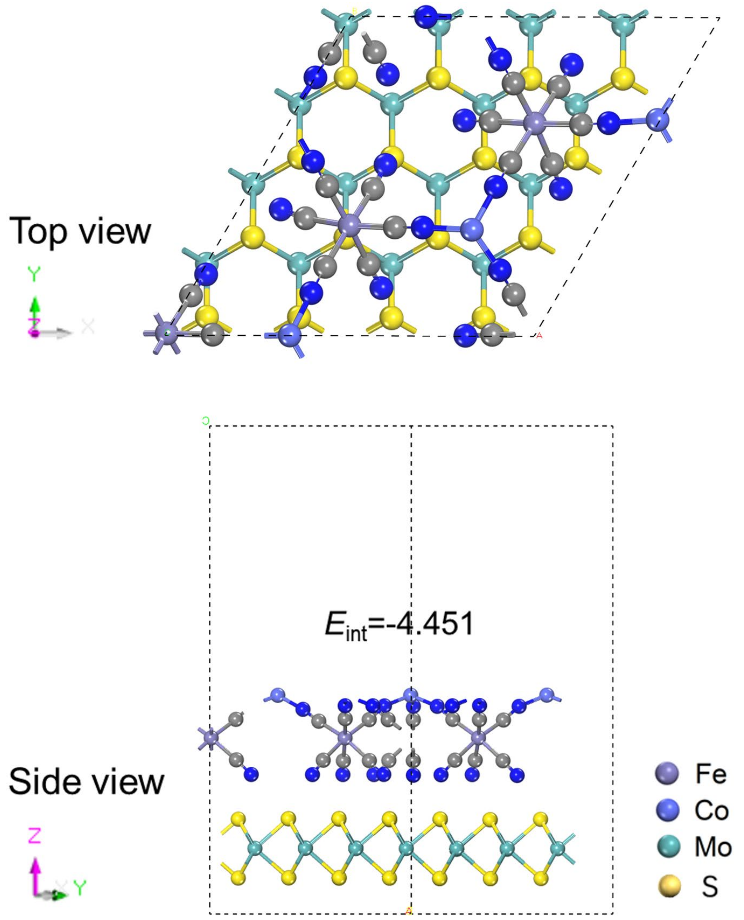


**Supplementary Figure 8. Crystal cell structures** of PBA/$MoS_2$ S–scheme heterojunction.

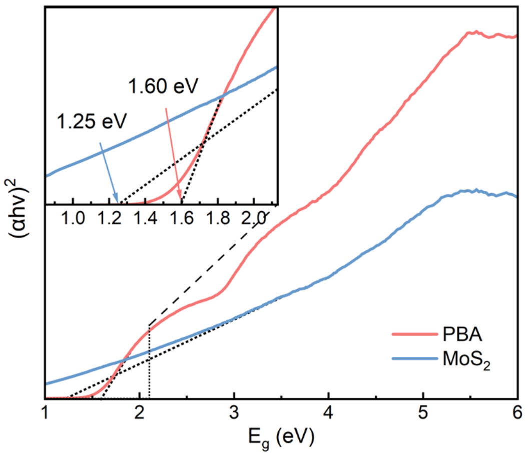


**Supplementary Figure 9. Kubelka–Munk energy curve plots** of as–prepared catalysts.

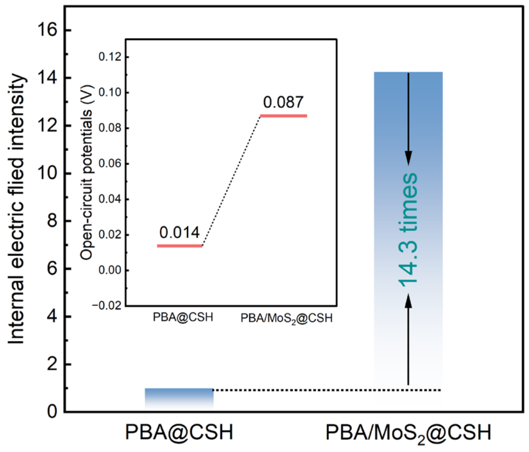


**Supplementary Figure 10. Open–circuit potentials (inset) and IEF intensity** of PBA@CSH and PBA/$MoS_2$@CSH. (Assuming the intensity of $Bi_2MoO_6$ to be 1).

**Supplementary Note 6.** The internal electric field (IEF) magnitude analysis

The internal electric field (IEF) magnitude of the PBA@CSH and PBA/$MoS_2$@CSH was calculated by using the following equation developed by Kanata et al [60, 61, 62, 63, 64]. It can be found from Supplementary Figure 9 that the IEF of PBA/$MoS_2$@CSH was 14.3 times as high as that of PBA@CSH.

$$F_S = (-2\rho V_S/\varepsilon\varepsilon_0)^{1/2}$$

$F_S$ is the IEF magnitude, $\rho$ is the surface charge density, $Vs$ is the surface voltage, $\varepsilon$ is the low–frequency dielectric constant, and $\varepsilon_0$ is the permittivity of free space, and $\varepsilon$ and $\varepsilon_0$ are two constants. That is, the IEF magnitude is mainly determined by $Vs$ and $\rho$. In order to evaluate their IEF magnitude, the surface charge densities by the transient photocurrent density measurements and surface voltages by open–circuit potential measurements were carefully surveyed, which are as shown in the Figure 2d and Supplementary Figure 9, respectively.

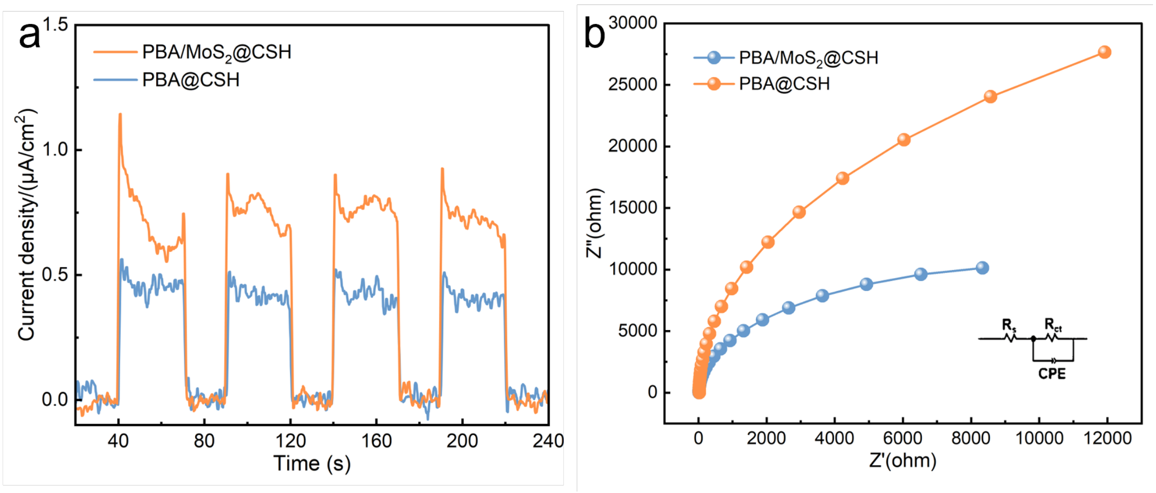


**Supplementary Figure 11. Electrochemical Characterization. a** Photocurrent response curves and **b** EIS Nyquist plots of PBA@CSH and PBA/$MoS_2$@CSH.

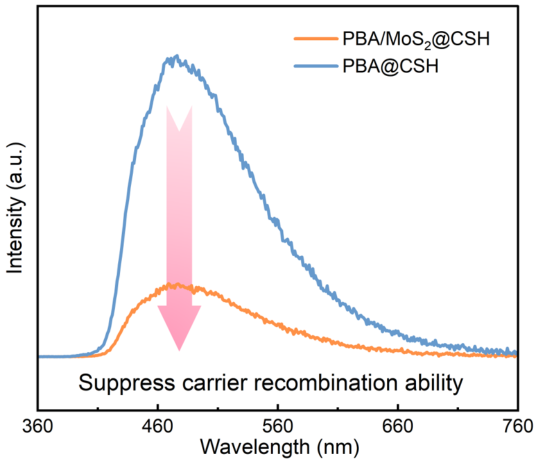


**Supplementary Figure 12. PL characterizations** of PBA@CSH and PBA/$MoS_2$@CSH, arrows indicate the direction of peak attenuation.

**Supplementary Table 3.** Exponential decay–fitted parameters of fluorescence lifetime.

| Samples | $\tau_1$ (ns) | $A_1$ | $\tau_2$ (ns) | $A_2$ | $\tau_{avg}$ (ns) |
|---|---|---|---|---|---|
| PBA@CSH | 1.294 | 141.966 | 5.269 | 17.756 | 2.635 |
| PBA/$MoS_2$@CSH | 1.348 | 390.577 | 6.542 | 75.994 | 3.871 |

**Supplementary Table 4.** Fitted parameters of the fs-TAS decay curves.

| Samples | $\tau_1$ (ps) | $A_1$ (%) | $\tau_2$ (ps) | $A_2$ (%) | $\tau_3$ (ps) | $A_3$ (%) | $\tau_{avg}$ (ps) |
|---|---|---|---|---|---|---|---|
| PBA@CSH | 5.762 | 0.308 (44.1) | 20.145 | 0.155 (22.2) | 310.494 | 0.236 (60.3) | 291.957 |
| PBA/$MoS_2$@CSH | 1.028 | 4.070 (85.2) | 52.844 | 0.399 (8.35) | 1322.480 | 0.310 (43.7) | 1248.318 |

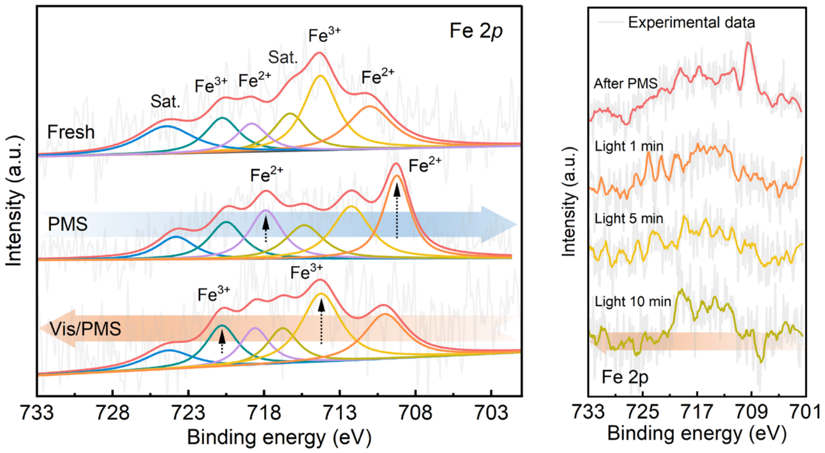


**Supplementary Figure 13. The high−resolution XPS spectra** of Fe 2*p* after PMS, light or Vis/PMS activation, orange and blue arrows indicate the direction of peak shift, respectively.

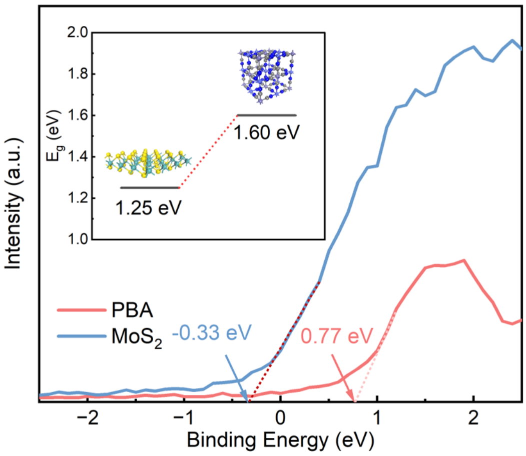


**Supplementary Figure 14. VB XPS spectra and the corresponding band gap** of as–prepared catalysts.

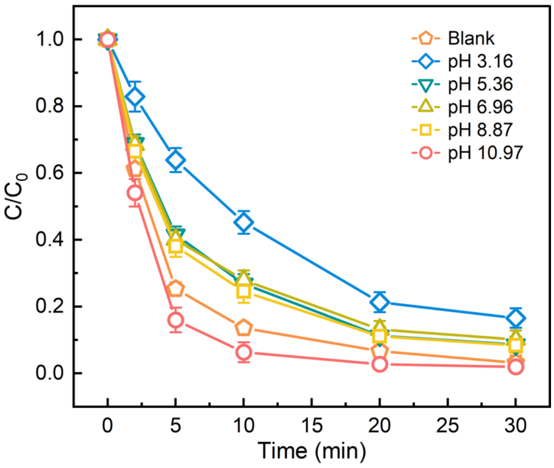


**Supplementary Figure 15. Catalytic degradation curve** of different pH with photoexcitation. Error bars represent the standard deviation of the experiment in triplicate.

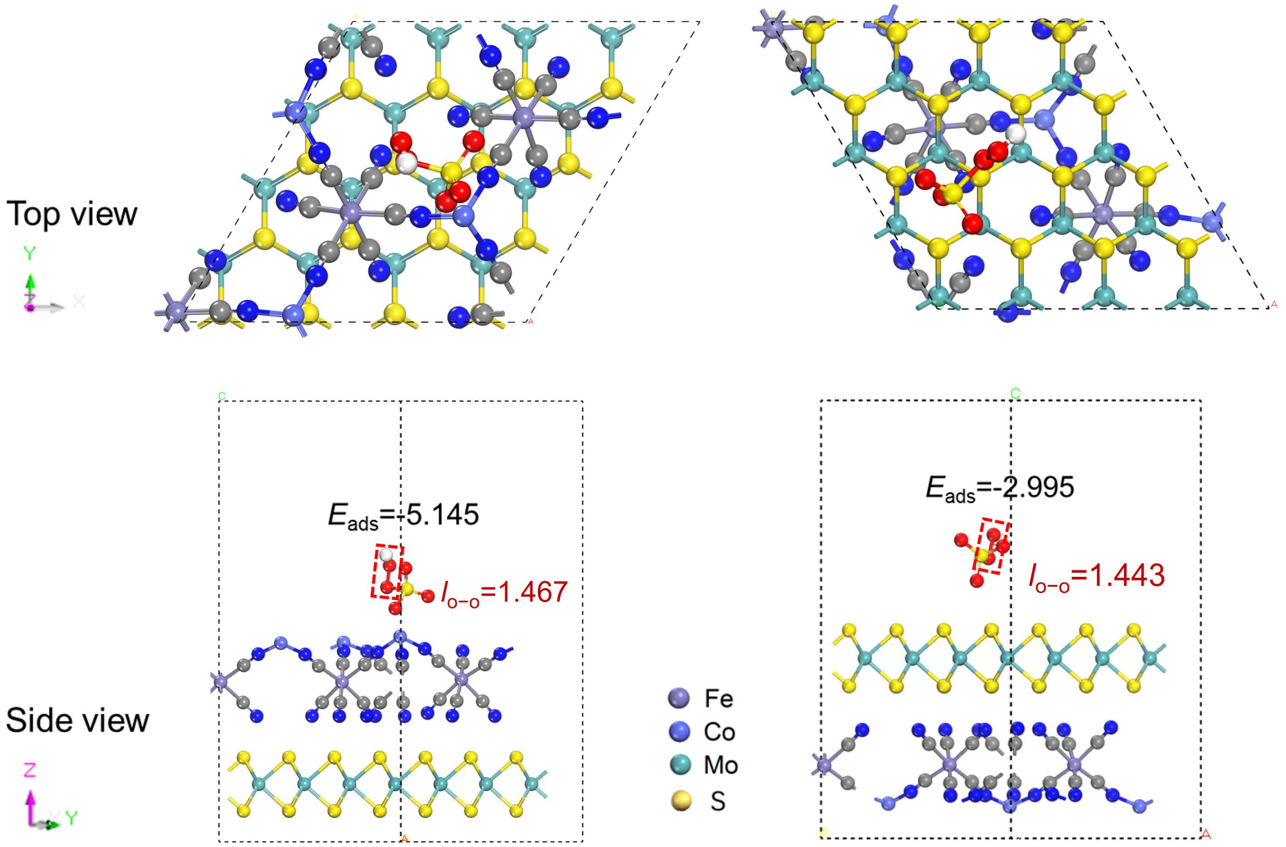


**Supplementary Figure 16. Crystal cell structures** of PMS on PBA/$MoS_2$ and $MoS_2$/PBA. PMS adsorbed at the PBA interface had longer O–O bonds and stronger $E_{ads}$, which indicates that the activation of PMS molecules on Co was more efficient.

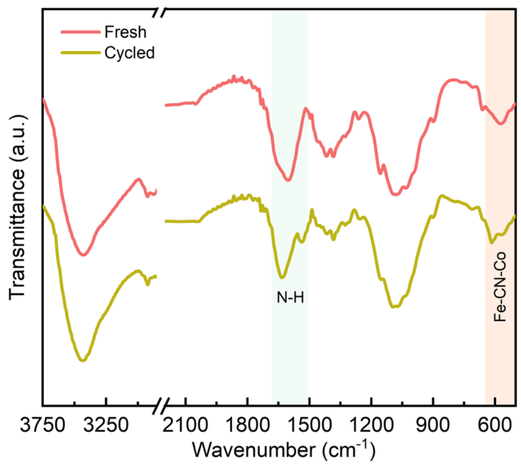


**Supplementary Figure 17. FTIR spectra** of PBA/$MoS_2$@CSH after cycled, orange and green highlights indicate the positions of functional group.

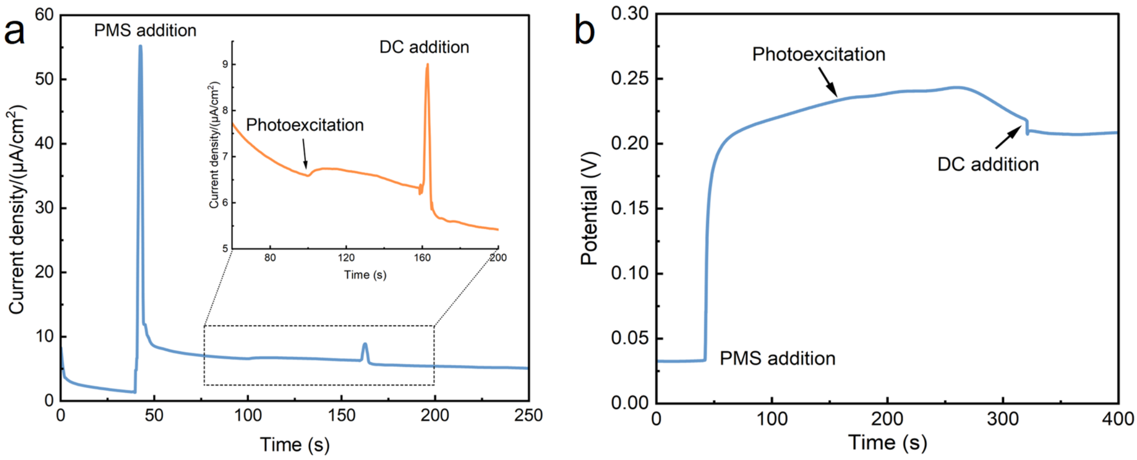


**Supplementary Figure 18. Electrochemical Characterization.** Chronoamperometry (a) and chronopotentiometry (b) curve of PBA/$MoS_2$@CSH with photoexcitation and adding PMS and DC.

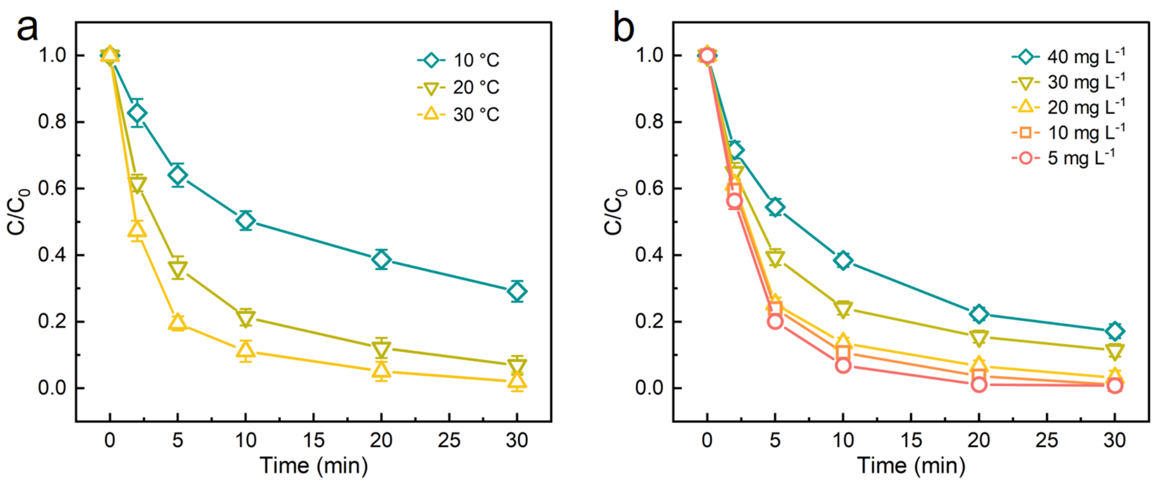


**Supplementary Figure 19. Catalytic degradation curve**. **a** Different temperature and **b** DC concentration. Error bars represent the standard deviation of the experiment in triplicate.

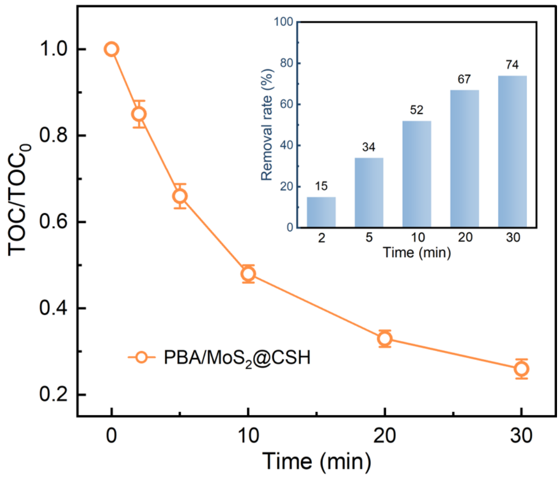


**Supplementary Figure 20. TOC removal ratio** during the degradation of DC by PBA/$MoS_2$@CSH. Error bars represent the standard deviation of the experiment in triplicate.

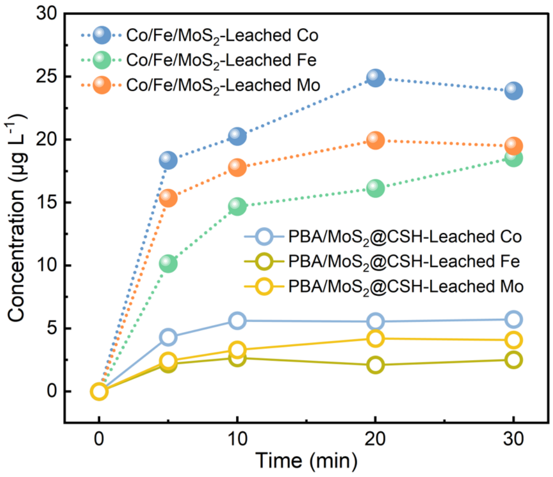


**Supplementary Figure 21. ICP analysis.** Leakage of Co, Fe and Mo elements into aqueous solution.

**Supplementary Table 5.** Leakage of ions (Co, Fe and Mo) of catalysts into aqueous solution after photocatalytic degradation of DC.

| Catalysts | Time (min) | Leached [Co] (μg L$^{-1}$) | Leached [Fe] (μg L$^{-1}$) | Leached [Mo] (μg L$^{-1}$) |
|---|---|---|---|---|
| Co/Fe/$MoS_2$ | 5 | 18.365 | 10.156 | 15.356 |
| | 10 | 20.254 | 14.689 | 17.786 |
| | 20 | 24.897 | 16.129 | 19.937 |
| | 30 | 23.871 | 18.554 | 19.499 |
| PBA/$MoS_2$@CSH | 5 | 4.31 | 2.19 | 2.432 |
| | 10 | 5.623 | 2.667 | 3.312 |
| | 20 | 5.559 | 2.108 | 4.21 |
| | 30 | 5.729 | 2.523 | 4.089 |

**Supplementary Table 6.** Comparison with other catalysts for the Metal leaching.

| Photocatalyst | Leaching concentration (μg $L^{-1}$) | | | |
|---|---|---|---|---|
| | Co | Fe | Mo | Reference |
| PBA/$MoS_2$@CSH | 5.729 | 2.523 | 4.089 | This work |
| Co/Fe/$MoS_2$ | 23.87 | 18.55 | 19.50 | Comparison |
| $MoS_2$/$CoFe_2O_4$ | 3462 | 2511 | / | [65] |
| $Co_3O_4$/$Bi_2MoO_6$ | 668.0 | / | / | [66] |
| sponge@$MoS_2$@GO | / | / | 180.0 | [67] |
| CoS@FeS | 394.5 | 303.7 | / | [68] |
| Co–CHNTs | 840.0 | / | / | [69] |
| $CoS_x$@$SiO_2$ | 560.0 | / | / | [70] |
| $Co_3O_4$–palygorskite | 449.0 | / | / | [71] |
| Co–NP | 270.0 | / | / | [72] |
| $NiCo_2O_4$NS | 810.0 | / | / | [73] |
| $FeCo_2S_4$–CN | 68.00 | 30.00 | / | [74] |
| CuS/MIL–Fe | / | 510.0 | / | [75] |
| Co@$MoS_2$–3 | 46.50 | / | / | [76] |
| $MoO_3$/$Bi_2O_3$/g–$C_3N_4$ | / | / | ~80.00 | [13] |
| $Fe_3O_4$@$CeO_2$@BiOI | / | 300.0 | / | [18] |
| FeNi–LDH@biochar | / | 120.0 | / | [77] |
| $Co_3O_4$/CPANI | 120.0 | / | / | [49] |
| CA–LDH | 354.2 | / | / | [55] |
| EGCG@$Fe_3O_4$ | / | 2200 | / | [58] |

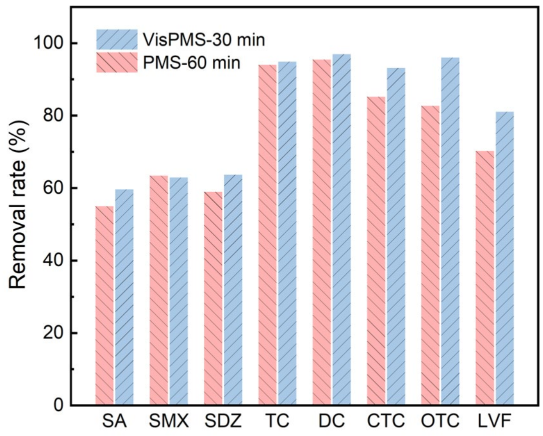


**Supplementary Figure 22. Degradation of three typical antibiotics**. Sulfonamides (SA, SMX and SDZ), tetracyclines (TC, DC, CTC and OTC) and quinolones (levofloxacin LVF) by PBA/$MoS_2$@CSH, respectively. Reaction Conditions: [concentration] = 20 mg $L^{-1}$, [catalyst] = 5 hydrogel spheres, [PMS] = 0.54 mM.

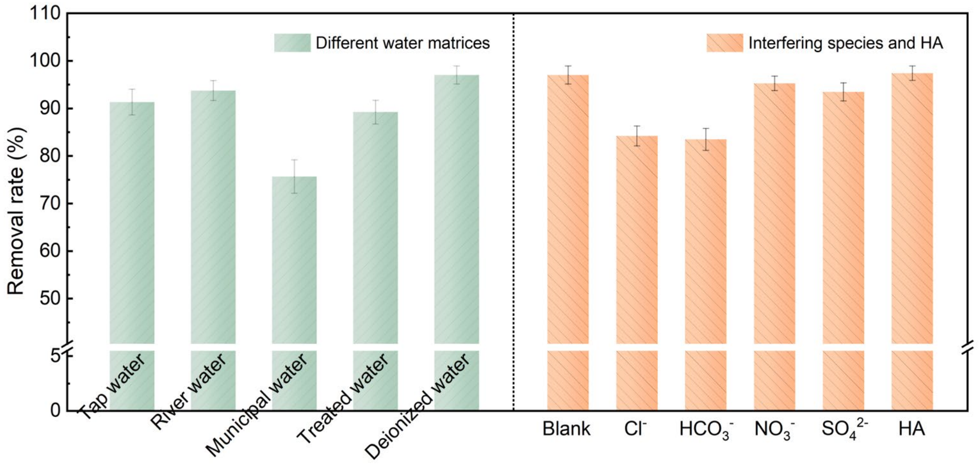


**Supplementary Figure 23. Effects of different water matrices** (Harbin, China) and anions $Cl^-$, $HCO_3^-$, $NO_3^-$, $SO_4^{2-}$ and HA on the DC degradation efficiencies. Error bars represent the standard deviation of the experiment in triplicate.

**Supplementary Note 7.** The degradation efficiency of DC by coexisting inorganic ions

The degradation efficiency and impact mechanism of DC were investigated. Humic acid (HA 2 mg $L^{-1}$) and inorganic anions such as $Cl^-$, $HCO_3^-$, $NO_3^-$ and $SO_4^{2-}$ were common in wastewater, and the corresponding salts were added to the reaction solution at a concentration of 10 mM. The degradation of DC by PBA/$MoS_2$@CSH was less affected by coexisting ions. The DC degradation was most affected by $Cl^-$ and $HCO_3^-$. This phenomenon could be explained by the quenching of $SO_4\cdot^-$ and ·OH by $Cl^-$ and $HCO_3^-$ to form reactive free radicals that cannot effectively oxidize DC, such as Cl·, $Cl_2\cdot^-$ and $ClO\cdot^-$, as well as low reactive carbonate radicals (i.e., $CO_3\cdot^-$ and $HCO_3\cdot$). In addition, $Cl^-$ and $HCO_3^-$ can directly consume PMS, which inhibits the activation process of PMS. $NO_3^-$ had little effect on the reaction. Interestingly, HA had a slight contribution to degradation, which had also been found in previous studies [78].

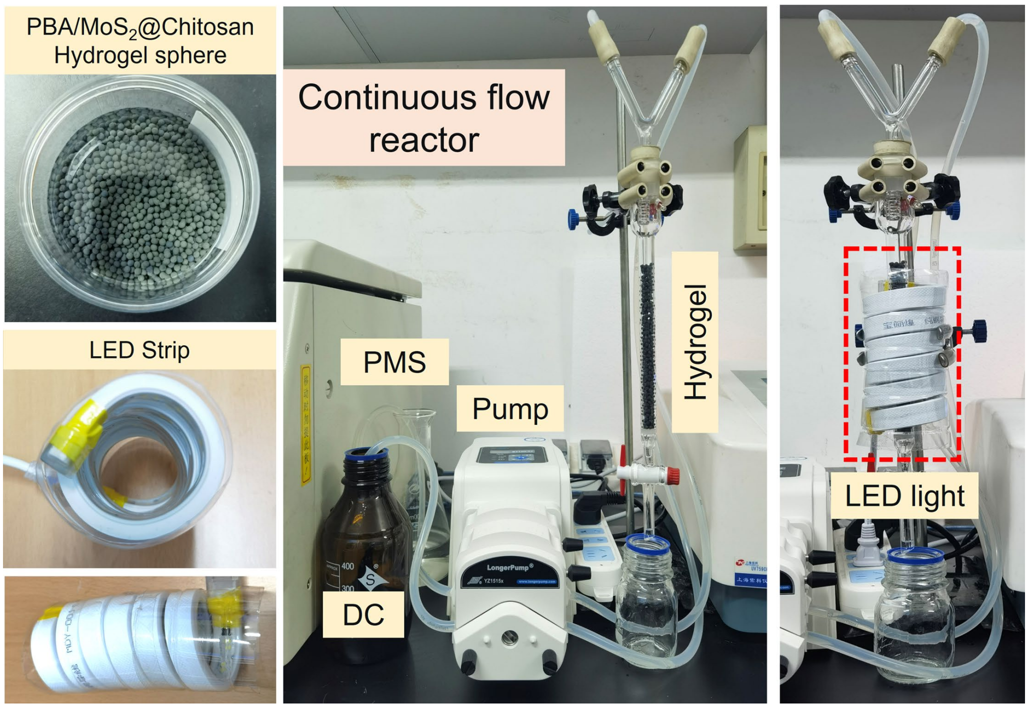


**Supplementary Figure 24. Continuous flow reaction device.** Photo of the LED photoexcitation catalytic column equipment. In the simulated continuous flow experiment, 5 mg $L^{-1}$ DC and 0.1g $L^{-1}$ PMS were injected into the top of the device at a flow rate of 2 mL $min^{-1}$ through a peristaltic pump. Samples were collected every 30min when the water flowed out from the bottom of the device.

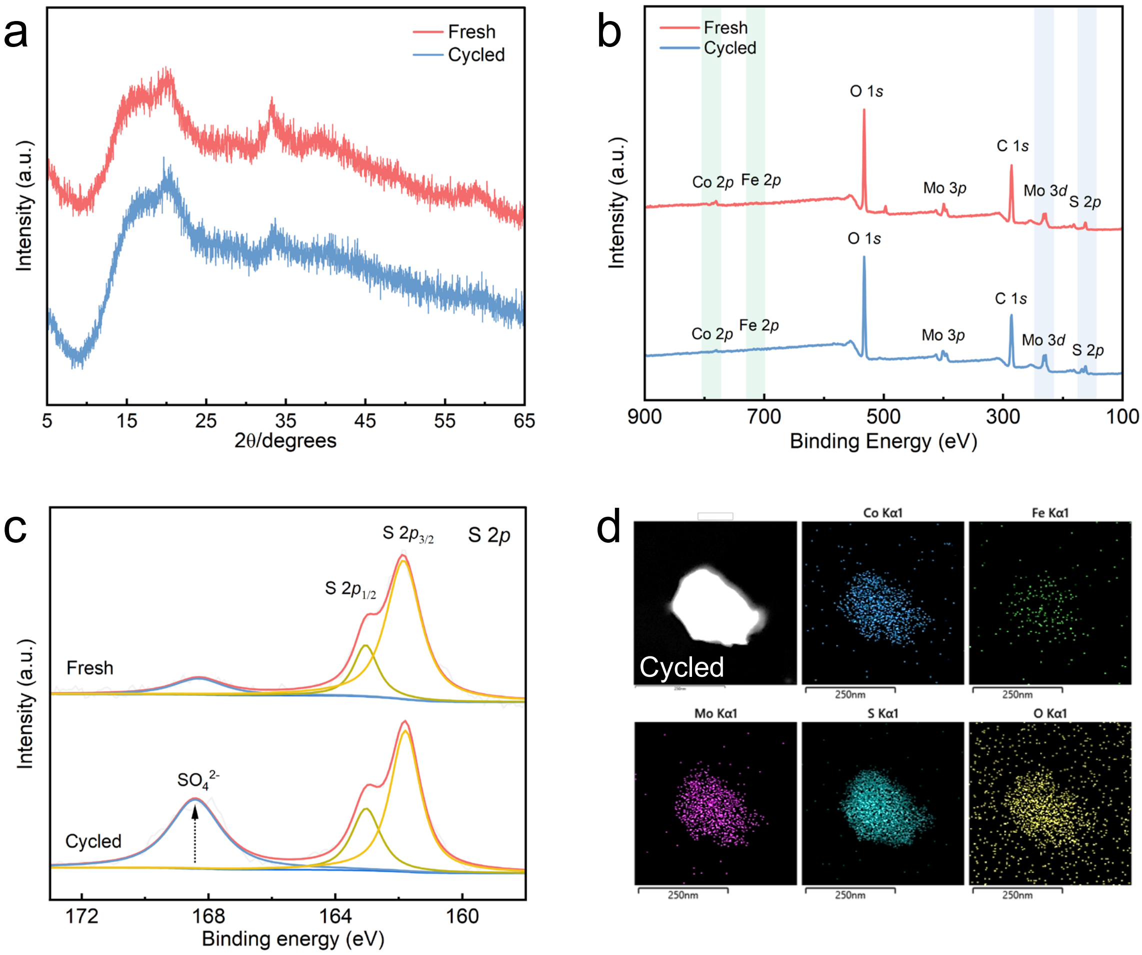


**Supplementary Figure 25. Stability analysis. a** The recycle of original and cycled XRD patterns, **b** XPS spectra and **c** the high−resolution XPS spectra of S 2*p* and **d** TEM mapping of PBA/$MoS_2$@CSH. The new appearance of the 168.4 eV peak attributed to S (VI) confirmed that $MoS_2$ was involved in the redox reaction and the formation of $SO_4^{2-}$ (Blue and green highlights indicate the positions of Mo 3*d*, S 2*p* and Co 2*p*, Fe 2*p*, respectively).

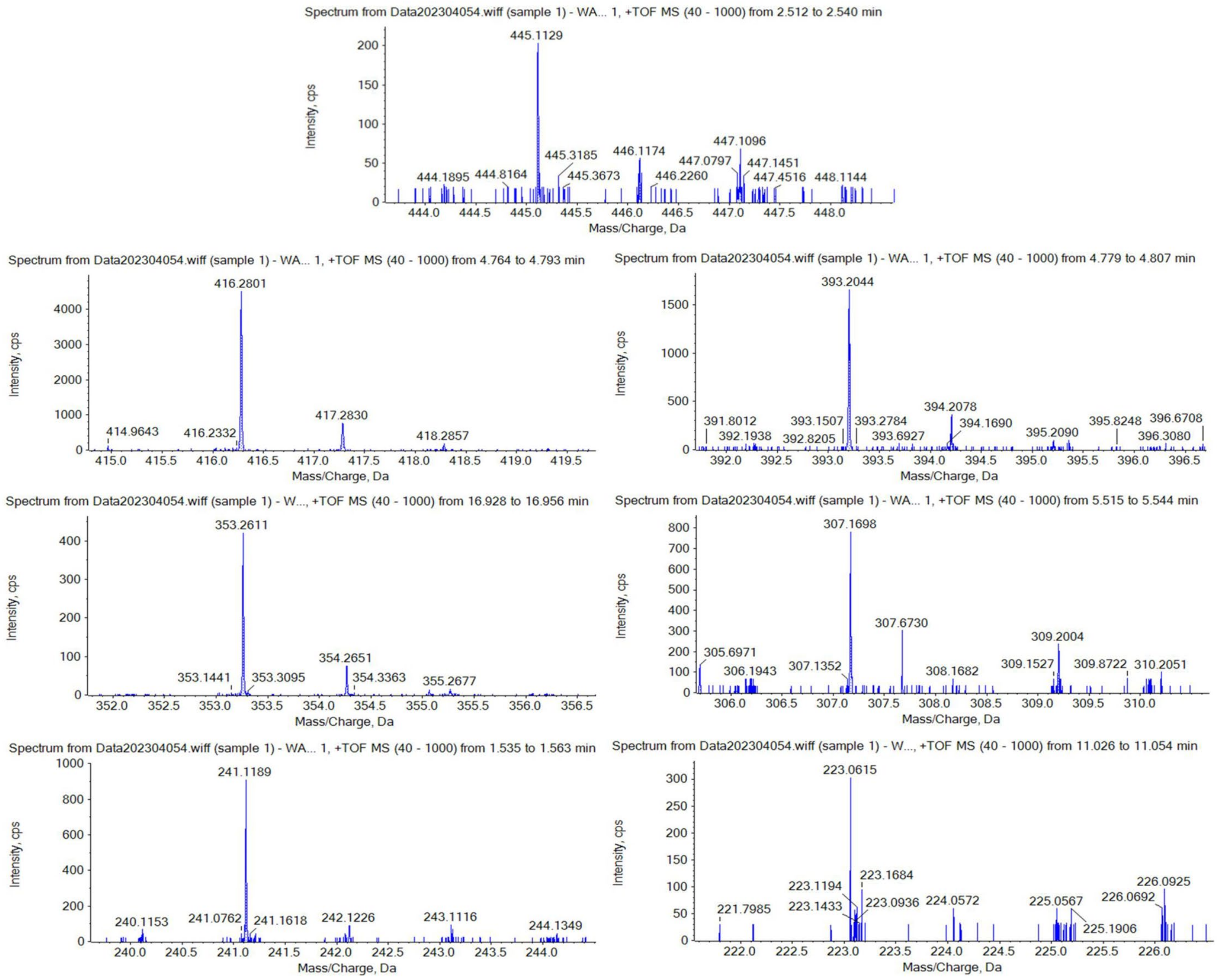


**Supplementary Figure 26. Fragment ions and oxidation products** in identification of DC in the catalytic system.

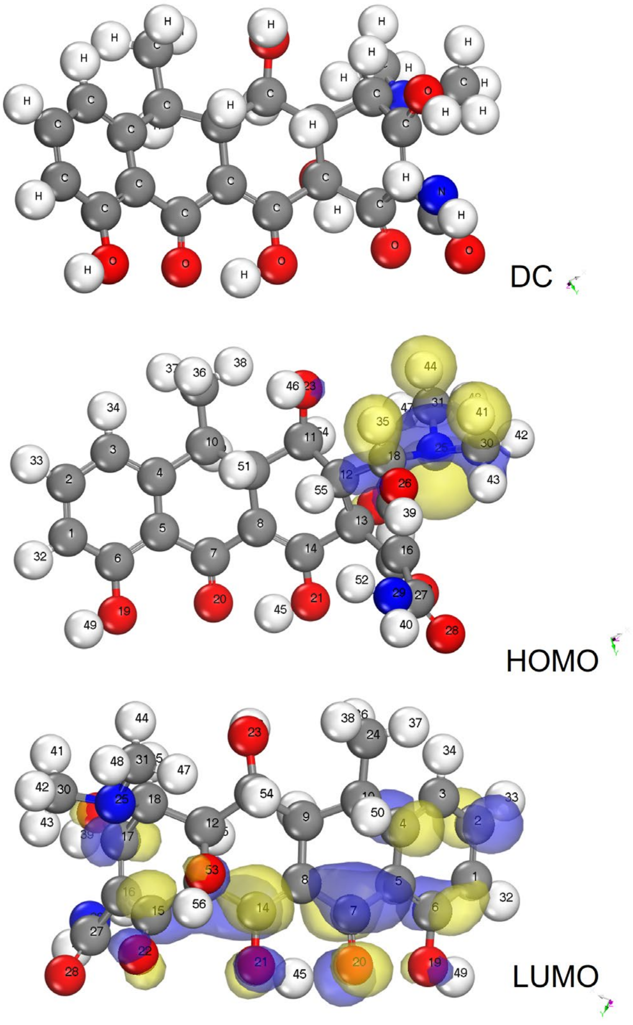


**Supplementary Figure 27. The HOMO and LUMO of DC**.

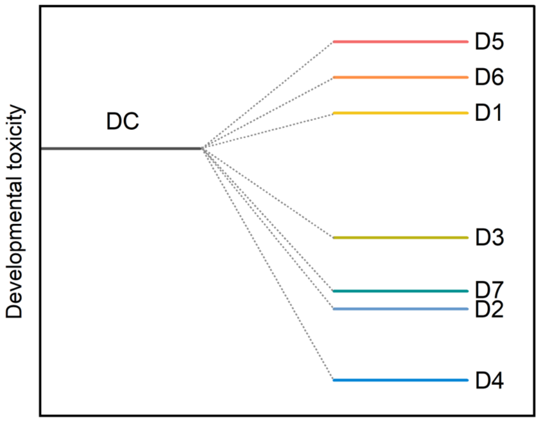


**Supplementary Figure 28. Theoretical calculated developmental toxicity** of DC and their degradation intermediates.

**Supplementary Table 7.** The structural information of the possible intermediate products.

| Name | Observed *m/z* | Formula | Proposed structure |
|---|---|---|---|
| **DC** | 445 | $C_{22}H_{24}N_2O_8$ | 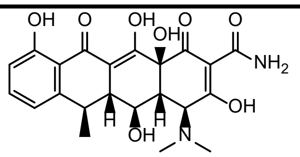 |
| **D1** | 416 | $C_{20}H_{17}NO_9$ | 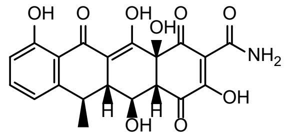 |
| **D5** | 417 | $C_{20}H_{20}N_2O_8$ | 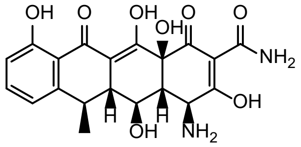 |
| **D6** | 353 | $C_{20}H_{16}O_6$ | 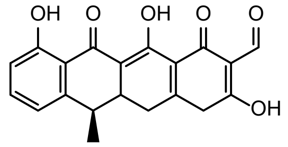 |
| **D2** | 393 | $C_{19}H_{20}O_9$ | 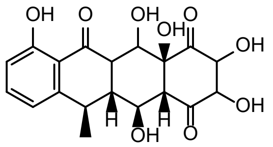 |
| **D3** | 307 | $C_{17}H_{22}O_5$ | 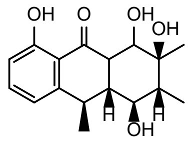 |
| **D4** | 241 | $C_{15}H_{12}O_3$ | 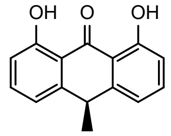 |
| **D7** | 223 | $C_{12}H_{14}O_4$ | 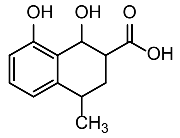 |

**Supplementary Table 8.** Calculated Fukui index of DC, highlighting indicates large peaks.

| No. | Atom | $f^-$ (Electrophilic) | $f^+$ (Nucleophilic) | $f^0$ (Radical) |
|---|---|---|---|---|
| 1 | C | 0.022 | 0.026 | 0.024 |
| 2 | C | 0.016 | 0.053 | 0.035 |
| 3 | C | 0.030 | 0.023 | 0.027 |
| 4 | C | 0.012 | 0.030 | 0.021 |
| 5 | C | 0.016 | 0.021 | 0.018 |
| 6 | C | 0.022 | 0.035 | 0.029 |
| 7 | C | 0.009 | 0.097 | 0.053 |
| 8 | C | 0.020 | 0.029 | 0.025 |
| 9 | C | 0.005 | 0.006 | 0.005 |
| 10 | C | 0.004 | 0.006 | 0.005 |
| 11 | C | 0.002 | 0.004 | 0.003 |
| 12 | C | 0.004 | 0.002 | 0.003 |
| 13 | C | 0.000 | 0.012 | 0.006 |
| 14 | C | 0.012 | 0.073 | 0.043 |
| 15 | C | 0.002 | 0.027 | 0.014 |
| 16 | C | 0.010 | 0.010 | 0.010 |
| 17 | C | 0.001 | 0.022 | 0.010 |
| 18 | C | 0.021 | 0.004 | 0.012 |
| 19 | O | 0.030 | 0.025 | 0.027 |
| 20 | O | 0.017 | 0.103 | 0.060 |
| 21 | O | 0.020 | 0.061 | 0.040 |
| 22 | O | 0.027 | 0.044 | 0.035 |
| 23 | O | 0.010 | 0.008 | 0.009 |
| 24 | C | 0.003 | 0.005 | 0.004 |
| 25 | N | 0.173 | 0.002 | 0.087 |
| 26 | O | 0.013 | 0.019 | 0.016 |
| 27 | C | 0.006 | 0.006 | 0.006 |
| 28 | O | 0.013 | 0.013 | 0.013 |
| 29 | N | 0.005 | 0.005 | 0.005 |
| 30 | C | 0.039 | 0.003 | 0.021 |
| 31 | C | 0.037 | 0.003 | 0.020 |

**Supplementary References:**


1. Gu L–L, *et al.* Thin–carbon–layer–enveloped cobalt–iron oxide nanocages as a high–efficiency sulfur container for Li–S batteries. *Journal of Materials Chemistry A* **8**, 20604–20611 (2020).
2. Zhao Q, Long M, Li H, Wen Q, Li D. Synthesis of $MFeO_3$/SBA–15 (M = La or Bi) for peroxymonosulfate activation towards enhanced photocatalytic activity. *New Journal of Chemistry* **46**, 1144–1157 (2022).
3. Liu M, *et al.* Confine activation peroxymonosulfate by surface oxygen vacancies of $BiO_{1-x}Cl$ to boost its utilization rate. *Separation and Purification Technology* **307**, 122711 (2023).
4. Kang J, *et al.* The enhanced peroxymonosulfate–assisted photocatalytic degradation of tetracycline under visible light by g–$C_3N_4$/Na–$BiVO_4$ heterojunction catalyst and its mechanism. *Journal of Environmental Chemical Engineering* **9**, 105524 (2021).
5. Chen R, Dou X, Xia J, Chen Y, Shi H. Boosting peroxymonosulfate activation over $Bi_2MoO_6$/$CuWO_4$ to rapidly degrade tetracycline: Intermediates and mechanism. *Separation and Purification Technology* **296**, 121345 (2022).
6. Jin C, *et al.* Two dimensional $Co_3O_4$/g–$C_3N_4$ Z–scheme heterojunction: Mechanism insight into enhanced peroxymonosulfate–mediated visible light photocatalytic performance. *Chemical Engineering Journal* **398**, 125569 (2020).
7. Tang R, *et al.* π–π stacking derived from graphene–like biochar/g–$C_3N_4$ with tunable band structure for photocatalytic antibiotics degradation via peroxymonosulfate activation. *Journal of Hazardous Materials* **423**, 126944 (2022).
8. Yi L, Li Y, Zhu L, Gao C, Wu X. CuO decorated natural rectorite as highly efficient catalyst for photoinduced peroxymonosulfate activation towards tetracycline degradation. *Journal of Cleaner Production* **317**, 128441 (2021).
9. Wang J, Jiang Y, Gao C, Li Y, Wu X. Synergistic effect of bimetal in three–dimensional hierarchical $MnCo_2O_4$ for high efficiency of photoinduced Fenton–like reaction. *Surfaces and Interfaces* **27**, 101482 (2021).
10. Li S, *et al.* NiO/g–$C_3N_4$ 2D/2D heterojunction catalyst as efficient peroxymonosulfate activators toward tetracycline degradation: Characterization, performance and mechanism. *Journal of Alloys and Compounds* **880**, 160547 (2021).
11. Shi H, He Y, Li Y, He T, Luo P. Efficient degradation of tetracycline in real water systems by metal–free g–$C_3N_4$ microsphere through visible–light catalysis and PMS activation synergy. *Separation and Purification Technology* **280**, 119864 (2022).
12. Jin C, Kang J, Li Z, Wang M, Wu Z, Xie Y. Enhanced visible light photocatalytic degradation of tetracycline by $MoS_2$/Ag/g–$C_3N_4$ Z–scheme composites with peroxymonosulfate. *Applied Surface Science* **514**, 146076 (2020).
13. Alnaggar G, Hezam A, Drmosh QA, Ananda S. Sunlight–driven activation of peroxymonosulfate by microwave synthesized ternary $MoO_3$/$Bi_2O_3$/g–$C_3N_4$ heterostructures for boosting tetracycline hydrochloride degradation. *Chemosphere* **272**, 129807 (2021).
14. Guo T, Jiang L, Huang H, Li Y, Wu X, Zhang G. Enhanced degradation of tetracycline in water over Cu–doped hematite nanoplates by peroxymonosulfate activation under visible light irradiation. *Journal of Hazardous Materials* **416**, 125838 (2021).
15. Liu Y, *et al.* Enhanced activation of peroxymonosulfate by a floating $FeMo_3O_x$/$C_3N_4$ photocatalyst under visible–light assistance for oxytetracycline degradation: Performance, mechanisms and comparison with $H_2O_2$ activation. *Environmental Pollution* **316**, 120668 (2023).
16. He Y, *et al.* Acceleration of levofloxacin degradation by combination of multiple free radicals via $MoS_2$ anchored in manganese ferrite doped perovskite activated PMS under visible light. *Chemical Engineering*

*Journal* **431**, 133933 (2022).

17. Zhou J, Liu W, Cai W. The synergistic effect of Ag/AgCl@ZIF–8 modified g–$C_3N_4$ composite and peroxymonosulfate for the enhanced visible–light photocatalytic degradation of levofloxacin. *Science of The Total Environment* **696**, 133962 (2019).
18. Kohantorabi M, Moussavi G, Oulego P, Giannakis S. Radical–based degradation of sulfamethoxazole via UVA/PMS–assisted photocatalysis, driven by magnetically separable $Fe_3O_4$@$CeO_2$@BiOI nanospheres. *Separation and Purification Technology* **267**, 118665 (2021).
19. Zhao J, Wang Y, Li N, Wang S, Yu J, Li X. Efficient degradation of ciprofloxacin by magnetic γ–$Fe_2O_3$–$MnO_2$ with oxygen vacancy in visible–light/peroxymonosulfate system. *Chemosphere* **276**, 130257 (2021).
20. Sivaranjani PR*, et al.* Fabrication of ternary nano–heterojunction via hierarchical deposition of α–$Fe_2O_3$ and β–$La_2S_3$ on cubic $CoCr_2O_4$ for enhanced photodegradation of doxycycline. *Journal of Industrial and Engineering Chemistry* **118**, 407–417 (2023).
21. Wen Q*, et al.* Synergetic effect of photocatalysis and peroxymonosulfate activated by Co/Mn–MOF–74@g–$C_3N_4$ Z–scheme photocatalyst for removal of tetracycline hydrochloride. *Separation and Purification Technology* **313**, 123518 (2023).
22. Vasanthakumar V*, et al.* α–$Bi_2(MoO_4)_3$ nanorods decorated with two–dimensional g–$C_3N_4$ nanosheets for efficient degradation of doxycycline under visible light illumination. *Process Safety and Environmental Protection* **163**, 1–13 (2022).
23. Pan Z, Qian L, Shen J, Huang J, Guo Y, Zhang Z. Construction and application of Z–scheme heterojunction $In_2O_3$/$Bi_4O_7$ with effective removal of antibiotic under visible light. *Chemical Engineering Journal* **426**, 130385 (2021).
24. Feng C*, et al.* Core–shell $Ag_2CrO_4$/N–GQDs@g–$C_3N_4$ composites with anti–photocorrosion performance for enhanced full–spectrum–light photocatalytic activities. *Applied Catalysis B: Environmental* **239**, 525–536 (2018).
25. Wang A*, et al.* MOF derived ZnO clusters on ultrathin $Bi_2MoO_6$ yolk@shell reactor: Establishing carrier transfer channel via PANI tandem S–scheme heterojunction. *Applied Catalysis B: Environmental* **328**, 122492 (2023).
26. Li D*, et al.* In–situ fabrication of ionic liquids/MIL–68(In)–$NH_2$ photocatalyst for improving visible–light photocatalytic degradation of doxycycline hydrochloride. *Chemosphere* **292**, 133461 (2022).
27. Pourmoslemi S, Mohammadi A, Kobarfard F, Amini M. Photocatalytic removal of doxycycline from aqueous solution using ZnO nano–particles: a comparison between UV–C and visible light. *Water science and technology : a journal of the International Association on Water Pollution Research* **74**, 1658–1670 (2016).
28. Zhang Z, Pan Z, Guo Y, Wong PK, Zhou X, Bai R. In–situ growth of all–solid Z–scheme heterojunction photocatalyst of $Bi_7O_9I_3$/g–$C_3N_4$ and high efficient degradation of antibiotic under visible light. *Applied Catalysis B: Environmental* **261**,  (2020).
29. Gao J, Gao Y, Sui Z, Dong Z, Wang S, Zou D. Hydrothermal synthesis of BiOBr/$FeWO_4$ composite photocatalysts and their photocatalytic degradation of doxycycline. *J Alloy Compd* **732**, 43–51 (2018).
30. Wang Q*, et al.* Unsaturated Nd–Bi dual–metal sites enable efficient NIR light–driven $O_2$ activation for water purification. *Applied Catalysis B: Environmental* **319**, 121924 (2022).
31. Jo W–K, Kumar S, Isaacs MA, Lee AF, Karthikeyan S. Cobalt promoted $TiO_2$/GO for the photocatalytic degradation of oxytetracycline and Congo Red. *Appl Catal B–Environ* **201**, 159–168 (2017).
32. Wei Z*, et al.* A novel 3D plasmonic p–n heterojunction photocatalyst: Ag nanoparticles on flower–like p–$Ag_2S$/n–$BiVO_4$ and its excellent photocatalytic reduction and oxidation activities. *Appl Catal B–Environ* **229**, 171–180 (2018).
33. Guan DL, Niu CG, Wen XJ, Guo H, Deng CH, Zeng GM. Enhanced Escherichia coli inactivation and

oxytetracycline hydrochloride degradation by a Z–scheme silver iodide decorated bismuth vanadate nanocomposite under visible light irradiation. *J Colloid Interf Sci* **512**, 272–281 (2018).

34. Wen XJ, Niu CG, Zhang L, Liang C, Zeng GM. An in depth mechanism insight of the degradation of multiple refractory pollutants via a novel $SrTiO_3$/BiOI heterojunction photocatalysts. *J Catal* **356**, 283–299 (2017).

35. Deng Y*, et al.* Construction of Plasmonic Ag and Nitrogen–Doped Graphene Quantum Dots Codecorated Ultrathin Graphitic Carbon Nitride Nanosheet Composites with Enhanced Photocatalytic Activity: Full–Spectrum Response Ability and Mechanism Insight. *Acs Appl Mater Inter* **9**, 42816–42828 (2017).

36. Hong Y*, et al.* Facile fabrication of stable metal–free CQDs/g–$C_3N_4$ heterojunctions with efficiently enhanced visible–light photocatalytic activity. *Sep Purif Technol* **171**, 229–237 (2016).

37. Jiang L*, et al.* Metal–free efficient photocatalyst for stable visible–light photocatalytic degradation of refractory pollutant. *Appl Catal B–Environ* **221**, 715–725 (2018).

38. Du Z*, et al.* Ultrathin h–BN/$Bi_2MoO_6$ heterojunction with synergetic effect for visible–light photocatalytic tetracycline degradation. *J Colloid Interface Sci* **589**, 545–555 (2021).

39. Yan M*, et al.* Fabrication of nitrogen doped graphene quantum dots–BiOI/$MnNb_2O_6$ p–n junction photocatalysts with enhanced visible light efficiency in photocatalytic degradation of antibiotics. *Appl Catal B–Environ* **202**, 518–527 (2017).

40. Liu J*, et al.* Conjugate Polymer–clothed $TiO_2$@$V_2O_5$ nanobelts and their enhanced visible light photocatalytic performance in water remediation. *Journal of Colloid and Interface Science* **578**, 402–411 (2020).

41. Ma R*, et al.* Enhanced catalytic degradation of aqueous doxycycline (DOX) in Mg–Fe–LDH@biochar composite–activated peroxymonosulfate system: Performances, degradation pathways, mechanisms and environmental implications. *Chemical Engineering Journal* **425**, 131457 (2021).

42. Luo X*, et al.* Green synthesis of manganese–cobalt‑tungsten composite oxides for degradation of doxycycline via efficient activation of peroxymonosulfate. *Journal of Hazardous Materials* **426**, 127803 (2022).

43. Luo X, Asefa T, Qiu R, Su C, Cui L, Huang Z. Robust Adsorption and Persulfate–Based Degradation of Doxycycline by Oxygen Vacancy–Rich Copper–Iron Oxides Prepared through a Mechanochemical Route. *ACS ES&T Water* **2**, 1031–1045 (2022).

44. Tang Y*, et al.* Catalytic degradation of oxytetracycline via $FeVO_4$ nanorods activating PMS and the insights into the performance and mechanism. *Journal of Environmental Chemical Engineering* **9**, 105864 (2021).

45. Wang A*, et al.* MOF Derived Co−Fe nitrogen doped graphite carbon@crosslinked magnetic chitosan Micro−nanoreactor for environmental applications: Synergy enhancement effect of adsorption−PMS activation. *Applied Catalysis B: Environmental* **319**, 121926 (2022).

46. Pang K*, et al.* Sulfur–modified chitosan derived N,S–co–doped carbon as a bifunctional material for adsorption and catalytic degradation sulfamethoxazole by persulfate. *Journal of Hazardous Materials* **424**, 127270 (2022).

47. Guo R, Wang Y, Li J, Cheng X, Dionysiou DD. Sulfamethoxazole degradation by visible light assisted peroxymonosulfate process based on nanohybrid manganese dioxide incorporating ferric oxide. *Applied Catalysis B: Environmental* **278**, 119297 (2020).

48. Liang J*, et al.* Persulfate Oxidation of Sulfamethoxazole by Magnetic Iron–Char Composites via Nonradical Pathways: Fe(IV) Versus Surface–Mediated Electron Transfer. *Environmental Science & Technology* **55**, 10077–10086 (2021).

49. Qian J*, et al.* Efficient emerging contaminants (EM) decomposition via peroxymonosulfate (PMS) activation by $Co_3O_4$/carbonized polyaniline (CPANI) composite: Characterization of tetracycline (TC) degradation property and application for the remediation of EM–polluted water body. *Journal of Cleaner Production* **405**, 137023 (2023).

50. Wang Y–q*, et al.* A novel partially carbonized $Fe_3O_4$@PANI–p catalyst for tetracycline degradation via

peroxymonosulfate activation. *Chemical Engineering Journal* **451**, 138655 (2023).

51. Hu Y, *et al.* Singlet oxygen–dominated activation of peroxymonosulfate by passion fruit shell derived biochar for catalytic degradation of tetracycline through a non–radical oxidation pathway. *Journal of Hazardous Materials* **419**, 126495 (2021).
52. Guo Y, *et al.* Goethite/biochar–activated peroxymonosulfate enhances tetracycline degradation: Inherent roles of radical and non–radical processes. *Science of The Total Environment* **783**, 147102 (2021).
53. Jiang H–L, *et al.* A novel oxygen vacancies enriched CoNi LDO catalyst activated peroxymonosulfate for the efficient degradation of tetracycline. *Journal of Water Process Engineering* **52**, 103526 (2023).
54. Liu L, *et al.* Insights into the performance, mechanism, and ecotoxicity of levofloxacin degradation in $CoFe_2O_4$ catalytic peroxymonosulfate process. *Journal of Environmental Chemical Engineering* **10**, 107435 (2022).
55. Qin L, *et al.* Citrate–regulated synthesis of hydrotalcite–like compounds as peroxymonosulfate activator – Investigation of oxygen vacancies and degradation pathways by combining DFT. *Applied Catalysis B: Environmental* **317**, 121704 (2022).
56. Jiang Z, *et al.* Electron transfer mechanism mediated nitrogen–enriched biochar encapsulated cobalt nanoparticles catalyst as an effective persulfate activator for doxycycline removal. *Journal of Cleaner Production* **384**, 135641 (2023).
57. Zhao Z, Wang P, Song C, Zhang T, Zhan S, Li Y. Enhanced Interfacial Electron Transfer by Asymmetric Cu–Ov–In Sites on $In_2O_3$ for Efficient Peroxymonosulfate Activation. *Angewandte Chemie* **135**, e202216403 (2023).
58. Tan C, *et al.* Activation of peroxymonosulfate by a novel EGCE@$Fe_3O_4$ nanocomposite: Free radical reactions and implication for the degradation of sulfadiazine. *Chemical Engineering Journal* **359**, 594–603 (2019).
59. Liu C, Wang Z, Hua S, Jiao H, Chen Y, Ding D. Sewage sludge derived magnetic biochar effectively activates peroxymonosulfate for the removal of norfloxacin. *Separation and Purification Technology* **314**, 123674 (2023).
60. Takashi K–K, Masayuki M, Hideyuki T, Yoshihiro H, Taneo N. Photoreflectance characterization of built–in potential in MBE–produced As–grown GaAs surface. In: *Proc.SPIE*) (1990).
61. Shi Y, *et al.* Coupled internal electric field with hydrogen release kinetics for promoted photocatalytic hydrogen production through employing carbon coated transition metal as co–catalyst. *Journal of Colloid and Interface Science* **630**, 274–285 (2023).
62. Chen Q, *et al.* Constructing oxide/sulfide in–plane heterojunctions with enlarged internal electric field for efficient $CO_2$ photoreduction. *Applied Catalysis B: Environmental* **297**, 120394 (2021).
63. Li J, Cai L, Shang J, Yu Y, Zhang L. Giant Enhancement of Internal Electric Field Boosting Bulk Charge Separation for Photocatalysis. *Advanced Materials* **28**, 4059–4064 (2016).
64. Chen X, Wang J, Chai Y, Zhang Z, Zhu Y. Efficient Photocatalytic Overall Water Splitting Induced by the Giant Internal Electric Field of a g–$C_3N_4$/rGO/PDIP Z–Scheme Heterojunction. *Advanced Materials* **33**, 2007479 (2021).
65. Feng S, *et al.* $MoS_2$/$CoFe_2O_4$ heterojunction for boosting photogenerated carrier separation and the dominant role in enhancing peroxymonosulfate activation. *Chemical Engineering Journal* **433**, 134467 (2022).
66. Guo J, *et al.* Highly efficient activation of peroxymonosulfate by $Co_3O_4$/$Bi_2MoO_6$ p–n heterostructure composites for the degradation of norfloxacin under visible light irradiation. *Separation and Purification Technology* **259**, 118109 (2021).
67. Zhu L, *et al.* Designing 3D–$MoS_2$ Sponge as Excellent Cocatalysts in Advanced Oxidation Processes for Pollutant Control. *Angewandte Chemie International Edition* **59**, 13968–13976 (2020).
68. Wu L, *et al.* The synergy of sulfur vacancies and heterostructure on CoS@FeS nanosheets for boosting the peroxymonosulfate activation. *Chemical Engineering Journal* **446**, 136759 (2022).
69. He Z, *et al.* Amorphous cobalt oxide decorated halloysite nanotubes for efficient sulfamethoxazole degradation

activated by peroxymonosulfate. *Journal of Colloid and Interface Science* **607**, 857–868 (2022).

70. Wang F*, et al.* Enhanced catalytic sulfamethoxazole degradation via peroxymonosulfate activation over amorphous $CoS_x$@$SiO_2$ nanocages derived from ZIF–67. *Journal of Hazardous Materials* **423**, 126998 (2022).

71. Yu Y, Ji Y, Lu J, Yin X, Zhou Q. Degradation of sulfamethoxazole by $Co_3O_4$–palygorskite composites activated peroxymonosulfate oxidation. *Chemical Engineering Journal* **406**, 126759 (2021).

72. Liu F*, et al.* Degradation of sulfamethoxazole by cobalt–nickel powder composite catalyst coupled with peroxymonosulfate: Performance, degradation pathways and mechanistic consideration. *Journal of Hazardous Materials* **400**, 123322 (2020).

73. Cai P*, et al.* Synergy between cobalt and nickel on $NiCo_2O_4$ nanosheets promotes peroxymonosulfate activation for efficient norfloxacin degradation. *Applied Catalysis B: Environmental* **306**, 121091 (2022).

74. Li Y*, et al.* Peroxymonosulfate activation on $FeCo_2S_4$ modified g–$C_3N_4$ ($FeCo_2S_4$–CN): Mechanism of singlet oxygen evolution for nonradical efficient degradation of sulfamethoxazole. *Chemical Engineering Journal* **384**, 123361 (2020).

75. Fang Z, Liu Y, Qi J, Xu Z–F, Qi T, Wang L. Establishing a high–speed electron transfer channel via CuS/MIL–Fe heterojunction catalyst for photo–Fenton degradation of acetaminophen. *Applied Catalysis B: Environmental* **320**, 121979 (2023).

76. Li X*, et al.* Effective removal of tetracycline from water by catalytic peroxymonosulfate oxidation over Co@$MoS_2$: Catalytic performance and degradation mechanism. *Separation and Purification Technology* **294**, 121139 (2022).

77. Mi X, Ma R, Pu X, Fu X, Geng M, Qian J. FeNi–layered double hydroxide (LDH)@biochar composite for , activation of peroxymonosulfate (PMS) towards enhanced degradation of doxycycline (DOX): Characterizations of the catalysts, catalytic performances,degradation pathways and mechanisms. *Journal of Cleaner Production* **378**, 134514 (2022).

78. Jia J, Liu D, Tian J, Wang W, Ni J, Wang X. Visible–light–excited humic acid for peroxymonosulfate activation to degrade bisphenol A. *Chemical Engineering Journal* **400**, 125853 (2020).
